# Dual Targeting Biomimetic Carrier‐Free Nanosystems for Photo‐Chemotherapy of Rheumatoid Arthritis via Macrophage Apoptosis and Re‐Polarization

**DOI:** 10.1002/advs.202406877

**Published:** 2025-01-22

**Authors:** Guanghe Xue, Huimei Jiang, Zhenhua Song, Yifan Zhao, Wen Gao, Bai Lv, Jie Cao

**Affiliations:** ^1^ Department of Pharmaceutics School of Pharmacy Qingdao University Qingdao 266071 China; ^2^ Department of Pharmacology School of Pharmacy Qingdao University Qingdao 266071 China; ^3^ Department of Radiation Oncology The Affiliated Hospital of Qingdao University Qingdao 266000 China

**Keywords:** carrier‐free, dual targeting, photo‐chemotherapy, repolarization, rheumatoid arthritis

## Abstract

Rheumatoid arthritis (RA) is a common chronic systemic autoimmune disease that often results in irreversible joint erosion and disability. Methotrexate (MTX) is the first‐line drug against RA, but the significant side effects of long‐term administration limit its use. Therefore, new therapeutic strategies are needed for treating RA. Here, dual‐targeting biomimetic carrier‐free nanomaterials (BSA‐MTX‐CyI nanosystem, BMC) is developed for synergistic photo‐chemotherapy of RA. Bovine serum albumin (BSA), which has high affinity with SPARC (secreted protein acidic and rich in cysteine) in the RA joint microenvironment, is selected as the hydrophilic end and coupled with MTX and the phototherapeutic agent CyI to self‐assemble into BMC. In vitro and in vivo experiments revealed that BMC accumulated significantly at the joint site in collagen antibody‐induced arthritis mice and could be specifically recognized and taken up by folate receptors in proinflammatory M1 macrophages. Upon near‐infrared laser irradiation, CyI exerted photodynamic and photothermal effects, whereas MTX not only inhibited cell proliferation but also increased cell sensitivity to reactive oxygen species, enhancing the apoptotic effect induced by CyI and achieving synergistic photo‐chemotherapy. Moreover, BMC could induce macrophages to reprogram into anti‐inflammatory M2 macrophages. This study provides innovative approaches for RA treatment via macrophage apoptosis and re‐polarization.

## Graphical abstract

In this manuscript, a dual‐targeting biomimetic carrier‐free nanosystem (BMC) is developed for synergistic photo‐chemotherapy of RA. Upon near‐infrared laser irradiation, BMC can not only synergistically kill anti‐inflammatory M1 macrophages, but also induce macrophages to reprogram into anti‐inflammatory M2 macrophages, as well as activate the cells’ own defense mechanism to express the anti‐inflammatory HSP70 protein.

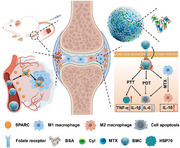

## 1 Introduction

Rheumatoid arthritis (RA) is one of the most common chronic autoimmune diseases, affecting approximately 1% of the global population. RA leads to persistent synovial and systemic inflammation, bone erosion, and cartilage damage, all of which seriously affect patients’ quality of life.^[^ ^1^, ^2^ ^]^ Methotrexate (MTX) is one of the most widely used first‐line drugs in the clinical treatment of RA, but its systematic administration often causes anemia, leukopenia, and other side effects.^[^ ^3^, ^4^, ^5^ ^]^ MTX is also prone to drug resistance, and the therapeutic dose needs to be gradually increased; despite this, approximately 50% of patients cannot achieve effective therapeutic effects due to drug resistance or the inability to tolerate its toxicity and side effects.^[^ ^6^, ^7^ ^]^ Current treatment methods often combine MTX with other drugs, such as glucocorticoids or biologics, but this combination treatment can only treat approximately 70% of patients, and the ideal state is to maintain low inflammation activity. Therefore, there is an urgent need to develop new therapeutic methods for RA as part of clinical treatment.

Phototherapy, which typically includes photodynamic therapy (PDT) and photothermal therapy (PTT), is recognized as one of the most promising modern medical technologies in the field of laser medicine. As a non‐invasive and safe treatment method, phototherapy has become one of the basic means of clinical treatment of tumor diseases.^[^ ^8^ ^]^ PDT refers to the use of photosensitizers to convert nearby oxygen molecules into cytotoxic reactive oxygen species (ROS), particularly singlet oxygen (^1^O_2_), which induces necrosis and apoptosis in surrounding cells.^[^ ^9^ ^]^ PTT uses photothermal transduction agents to absorb light energy and convert it into heat energy, causing irreversible thermal damage to surrounding cells and tissues.^[^ ^10^ ^]^ RA synovial tissues exhibit rapid, malignant proliferation, strong metabolism, and aggressiveness, which are similar to the pathological environment fostering tumor cell growth.^[^ ^11^, ^12^ ^]^ Thus, researchers have made great efforts to use phototherapeutic agents to treat RA, and phototherapy has shown broad clinical development prospects for the treatment of RA.^[^ ^13^, ^14^, ^15^ ^]^ In addition, current research has found that mild hyperthermia can promote bone growth, and that heat‐activated heat shock proteins such as heat shock proteins 70 (HSP70), which can be generated during PTT, can fight inflammation by affecting the NF‐κb pathway to downregulate the production of pro‐inflammatory factors.^[^ ^16^, ^17^, ^18^ ^]^ In addition, most of the photosensitizers used in phototherapy can be employed in fluorescence imaging, which can compensate for deficiencies in the current clinical diagnosis of RA.^[^ ^19^ ^]^ However, most photosensitizers and photothermal transduction agents have the disadvantages of poor water solubility, lack of targeting, and low bioavailability at the target site; therefore, it is essential to efficiently and specifically deliver therapeutic agents to site of RA to improve its therapeutic index and reduce systemic toxicity.

Due to the infiltration of inflammatory cells at the site of RA infection, angiogenesis is promoted, and sufficient nutrients are transported to the inflamed area, leading to rapid growth of synovial tissue.^[^ ^20^ ^]^ The new tissue fills a wide gap (up to 700 nm) at the junction between endothelial cells. Thus, it is expected that appropriately sized nanoparticle carriers can passively diffuse and accumulate within the synovial tissue space between endothelial cells, producing the so‐called “ELVIS” effect (through extravasation of the leaky vasculature and subsequent inflammatory cell‐mediated isolation), which is similar to the enhanced permeability and retention (EPR) effect observed in solid tumors.^[^ ^21^ ^]^ Therefore, various nanomaterials, including liposomes, dendrimers, micelles, and inorganic nanoparticles, have been developed to deliver therapeutic agents to the inflammatory sites of RA.^[^ ^22^, ^23^, ^24^, ^25^ ^]^ For example, Juillerat‐Jeanneret et al. incorporated three anionic photosensitizers (TPPS_4_, TPPC_4_, and Ce6) into a chitosan nanogel modified with hyaluronic acid to increase the retention time of the drug in the joint and achieve more effective PDT for treating RA.^[^ ^26^ ^]^ Gao et al. designed a metal/semiconductor composite consisting of an octahedral copper sulfide shell and a gold nanorod core (Au NR@CuS) for the combined treatment of RA with PTT, PDT, and chemotherapy.^[^ ^27^ ^]^ Although these developments have remarkable merits, translating this basic research to a viable market product remains challenging. Particularly, nano‐delivery systems are typically inert, acting only as vehicles, but their presence can increase toxicity to normal tissues, and their subsequent degradation can invoke an in vivo immune reaction, imposing an extra burden on patients. Poor delivery efficiency is another concern that has hindered effective clinical trials.

Recently, carrier‐free delivery systems, in which pure drug nanoparticles act as carriers, have been proposed as attractive nanomedicines due to advantages such as high biosafety, large drug loading, simple drug composition, and simplified synthesis process.^[^ ^28^, ^29^ ^]^ Such systems do not require any external inert agents, and they have the following advantages: 1) High biosafety, no toxicity, and no immunogenicity caused by the carrier; 2) the drug composition and preparation procedure are simple, and the loading rate is high; and 3) the nanoscale characteristics of the drug itself can achieve the targeted effect and increase the accumulated concentration of the drug at the action site.

**Figure 1:**
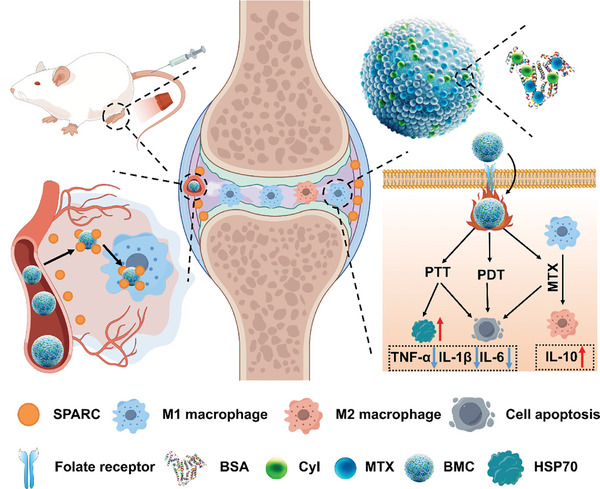
Schematic of bionic carrier‐free nanosystems with double‐targeted pathways and photo‐chemotherapy for RA. SPARC: secreted protein acidic and rich in cysteine; BSA: bovine serum albumin; MTX: methotrexate; BMC: BSA‐MTX‐CyI nanosystem; HSP70: heat shock proteins 70.

Inspired by the above concerns, we adopted a simple and green self‐assembly method to develop a dual‐targeting carrier‐free nanosystem (BSA‐MTX‐CyI, BMC) involving the hydrophilic targeting ligand BSA, hydrophobic anti‐rheumatic drug MTX, and the phototherapeutic reagent CyI (**Figure** **1**). CyI, an iodinated cyanine dye, which was synthesized in our previous study by integrating heavy atom I into the cyanine dye Cy7, exhibits an enhanced singlet oxygen yield (75%) due to the heavy atom effect. CyI also exhibits high photo‐to‐photothermal conversion efficiency and near‐infrared (NIR) absorption, making it particularly useful not only as a photosensitizer for PDT, but also as a photothermal agent for PTT, as well as an NIR imaging agent.^[^ ^30^, ^31^, ^32^, ^33^, ^34^ ^]^ MTX is a folate analog with structural similarity to folic acid; consequently, its pharmacological action depends on competing with folate for the dihydrofolate reductase site.^[^ ^35^ ^]^ Therefore, MTX is not only valuable as an anti‐rheumatic drug but also has a high affinity for the folate receptor overexpressed in M1 macrophages, which can act as a targeting ligand to guide better uptake of BMC by inflammatory macrophages. Previous studies have shown that the glycoprotein SPARC (secreted protein acidic and rich in cysteine; also known as osteonectin), a member of the family of matricellular components of the extracellular matrix (ECM), is overexpressed in the synovial fluid and joint tissues of patients with RA, which have high affinity for albumin.^[^ ^36^ ^]^ In addition, due to the increased metabolism of synovial cells during RA, arthritic joints metabolize higher amounts of albumin than healthy tissues, using it as a nitrogen and energy source, resulting in an increased demand for albumin in arthritic joints.^[^ ^37^ ^]^ Hence, we selected BSA as another raw material and targeting ligand to boost the delivery efficiency of the albumin‐based nanosystem to inflammatory arthritis. Under NIR irradiation, CyI undergoes transitions to singlet electronic states that decay by ^1^O_2_ and photothermal pathways that inflict PDT‐ and PTT‐induced damage mechanisms to M1 macrophages at the lesion area. Meanwhile, phototherapy can accelerate the release of MTX, achieving timing‐ and light‐controlled drug delivery, as well as polarizing proinflammatory M1 macrophages into anti‐inflammatory M2 macrophages to achieve synergistic photo‐chemotherapeutic anti‐rheumatic therapy. In addition, the mild PTT effect can promote bone growth and upregulate the expression of osteoblast protein, while HSP70 produced by heat activation also have anti‐inflammatory effects and can downregulate the expression of inflammatory factors.^[^ ^38^, ^39^, ^40^, ^41^ ^]^ This study provides preliminary in vitro and in vivo evidence in an RA mouse model, which suggests that dual‐targeting carrier‐free nanosystems hold promise for RA treatment.

## 2 Results and Discussion

### 2.1 Preparation and Characterization of a Dual‐Targeting Carrier‐Free BMC Nanosystem

**Figure 2:**
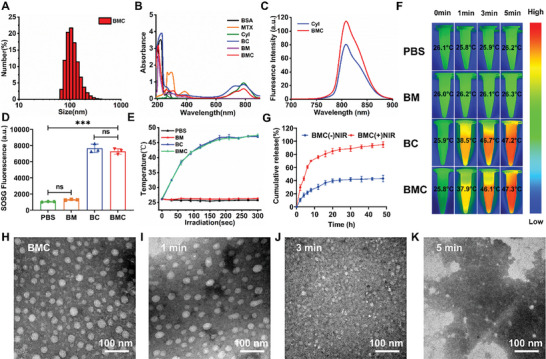
Characterization of BMC nanosystems. (A) Hydrodynamic diameter of BMC. (B) UV–Vis‐NIR absorption spectra of BSA, MTX, CyI, BC, BM, and BMC. (C) Fluorescence spectra of CyI and BMC (Ex: 780 nm). (D) Singlet oxygen production by PBS, BM, BC, and BMC under NIR irradiation (808 nm, 0.96 W cm^−2^, 5 min) (*n* = 3); ***p < 0.001. (E) Temperature change curves of PBS, BM, BC, and BMC upon laser irradiation (0.96 W cm^−2^, 808 nm, 5 min) (*n* = 3). (F) NIR thermal images of different samples at different times (0, 1, 3, and 5 min). (G) Accumulative MTX release curve from BMC with or without an “on‐off” NIR light (0.96 W cm^−2^, 808 nm) for 5 min at 37 °C (*n* = 3). (H) TEM image of BMC (scale bar = 100 nm); TEM images of BMC continuously irradiated with NIR laser (0.96 W cm^−2^, 808 nm) for 1 min (I), 3 min (G), and 5 min (K) (scale bar = 100 nm).

A carrier‐free nanosystem for co‐delivery of the photosensitizer and anti‐rheumatic drug to the inflammatory site was prepared via self‐assembly methods. CyI, a hydrophobic iodinated cyanine dye, was employed as the photosensitizer/photothermal/and NIR imaging agent but lacks targeting capabilities. MTX, an anti‐rheumatic drug with active targeting properties that targets activated M1 macrophages via FRβ receptors, also has poor water solubility. Hence, we used BSA as a hydrophilic block to enhance the loading capacity of CyI and MTX, extend their retention times at the synovial joint, and prevent CyI from self‐aggregation in circulating blood. Simply, BSA‐MTX‐CyI (BMC) was formed via reactions between the carboxyl groups of MTX and CyI and the amino groups of BSA. BMC nanosystems were then obtained by a simple green self‐assembly method. The obtained BMC exhibited a uniform diameter distribution with a size of 111.48 ± 5.95 nm, and a polydispersity index (PDI) of 0.13 ± 0.015 (**Figure** **2**A). The zeta potential was −17.13 ± 0.11 mV, indicating good blood circulation stability and low electrostatic attraction to cell membranes. The transmission electron microscopy (TEM) images in Figure 2H show that the resulting BMC nanosystem was uniform in size and exhibited a spherical structure. Note that the size measured by dynamic light scattering (DLS) was significantly larger than that measured by TEM, possibly due to shrinkage of BMC during TEM sample preparation. As shown in Figure 2B, the characteristic peaks of 220 nm (BSA), 306 nm (MTX), and 798 nm (CyI) can be clearly observed in the absorption spectra of BMC, indicating the successful formation of BMC. As shown in Figure 2C, BMC and CyI have the same fluorescence emission peak (≈810 nm) under 780 nm excitation light, indicating that BMC excited in the NIR region can be used for fluorescence imaging. Next, the stability of BMC stored in water, phosphate‐buffered saline (PBS), and DMEM at ambient temperature for 14 days was investigated by DLS. As shown in Figure S1 (Supporting Information), the size of the sample did not change significantly, indicating that BMC exhibits good stability in different media. In addition, the stability of BMC placed in DMEM containing 10% fetal bovine serum (FBS) in a simulated serum medium was monitored for 48 h, and no significant changes in diameter were observed (Figure S2, Supporting Information). These results indicate that BMC nanosystems exhibit good physical stability.

In previous studies, we confirmed that CyI exhibits good photodynamic and photothermal effects under NIR irradiation.^[^ ^30^ ^]^ Here, SOSG, a singlet oxygen sensor green, was used to determine the singlet oxygen production capacity of BMC. The results shown in Figure 2D demonstrate that after 5 min of NIR irradiation (808 nm, 0.96 W cm^−2^), essentially no ^1^O_2_ was produced in the PBS and BM groups, whereas a large amount of ^1^O_2_ was generated in both the BC and BMC groups, indicating that the in vitro PDT effect of BMC was mainly dependent on CyI. Current studies have validated the potential of mild PTT for the treatment of RA.^[^ ^42^, ^43^ ^]^ Therefore, we studied the photothermal conversion ability of BMC under NIR irradiation. The results depicted in Figure 2E demonstrate that upon NIR irradiation for 5 min, the temperatures of the PBS and BM groups remained relatively stable. Conversely, the temperature of the BMC and BC groups increased by approximately 20 °C within 3 min, resulting in a sustained but mild PTT effect. The thermal imaging images (Figure 2F) confirm the excellent photothermal‐conversion capability of BMC.

Next, the real‐time release profile of MTX under NIR irradiation was monitored for 48 h and plotted in Figure 2G. As shown, MTX in BMC reached complete release (>80%) at 20 h after NIR irradiation for 5 min, compared to only 37% release at 20 h in unirradiated BMC, demonstrating that the nanostructure could be disrupted under NIR irradiation. The degradation of BMC was then monitored using TEM. As shown in Figure 2I–K, when BMC was irradiated by an NIR laser (808 nm, 0.96 W cm^−2^) for 1 min, the nanosystem maintained a relatively complete structure, but after 3 min of irradiation, the morphology of BMC began to change. After 5 min of irradiation, most of the particles lost their spherical structure and basically disintegrated completely. These results confirm our hypothesis that BMC can be degraded due to the PTT effect after laser irradiation, which may lead to the rapid release of drugs at the target site, highlighting its value as an NIR light‐triggered nanosystem for drug release.

### 2.2 In Vitro Targeting of BMC to M1 Macrophages

MTX is a folate analog with a chemical structure similar to that of folate and has been reported to have high affinity for folate receptors in tumor cells.^[^ ^44^, ^45^, ^46^ ^]^ We previously verified that folate receptors are highly expressed in M1 macrophages compared to other cells in the inflammatory environment of RA.^[^ ^34^ ^]^ Therefore, we assume that BMC can target M1‐type macrophages. Before evaluating this hypothesis, we sought to establish and affirm models of M1‐ and M2‐like activated macrophages by stimulating initial murine monocyte‐derived RAW264.7 cells (M0‐like) with either lipopolysaccharide (LPS; M1‐like) or interleukin‐4 (IL‐4; M2‐like). As shown in Figures S3 and S4 (Supporting Information), the fluorescence signals of the M1‐type macrophage biomarker iNOS or the M2‐type macrophage biomarker CD206 were significantly increased by stimulation with LPS or IL‐4, respectively, compared to their expression in RAW 264.7 macrophages, confirming that RAW 264.7 cells differentiated into M1 or M2 macrophages after LPS or IL‐4 stimulation. In parallel, we stimulated the differentiation of human monocyte leukemia cells THP‐1 into M0‐like macrophages through phorbol 12‐myristate 13‐acetate (PMA). M0‐like macrophages differentiated from THP‐1 were stimulated to polarize into M1‐ or M2‐like macrophages by adding LPS + interferon‐γ (IFN‐γ) or interleukin‐3 (IL‐3) + interleukin‐14 (IL‐14). As shown in Figures S5 and S6 (Supporting Information), iNOS expression in THP‐1‐differentiated M1‐like macrophages increased significantly after stimulation with LPS+IFN‐γ. After stimulation with IL‐3+IL‐14, the expression of CD206 was significantly increased in M2‐like macrophages differentiated from THP‐1. These experiments confirm that M0‐like macrophages derived from THP‐1 could be polarized into M1‐ or M2‐like macrophages following LPS + IFN‐γ or IL‐3 + IL‐14 stimulation.

**Figure 3:**
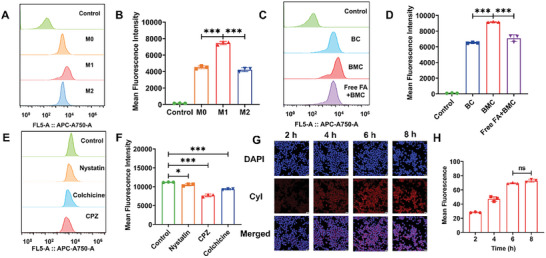
In vitro targeting ability and cellular uptake of BMC by M1 macrophages. (A) The intracellular fluorescence intensity of different cells incubated with BMC (CyI concentration of 45 µg mL^−1^) for 6 h was determined by flow cytometry. (B) The fluorescence intensity in (A) was quantified using FlowJo software. (C) The intracellular fluorescence of M1 macrophages was determined by flow cytometry after incubation with PBS, BC, BMC, or FA+BMC (CyI concentration is 45 µg mL^−1^) for 6 h. (D) The fluorescence intensity in (C) was quantified using FlowJo software. (E) The intracellular fluorescence of M1 macrophages was determined by flow cytometry after incubation with BMC for 6 h after treatment with different endocytosis inhibitors. (F) The fluorescence intensity in (E) was quantified using FlowJo software. (G) Confocal fluorescence images of M1 macrophages cultured with BMC (CyI concentration of 45 µg mL^−1^) for 2, 4, 6, and 8 h; scale bar: 20 µm. (H) Semiquantitative fluorescence analysis of M1 macrophages cultured with BMC for different times. All data are presented as the mean ± SD, *n* = 3. *p < 0.05, ***p < 0.001, NS: Not significant.

Next, to verify the specificity of BMC to M1 macrophages, BMC was incubated with different types of macrophages (M0, M1, and M2 types) for the same time, before investigating the targeting properties by flow cytometry. As shown in **Figure** **3**A,B, the amount of BMC uptake by M1 macrophages was much higher than that of M0 and M2 macrophages. The confocal laser scanning microscopy (CLSM) images and semi‐quantitative results showed the same results (Figure S7, Supporting Information), demonstrating that BMC is preferentially taken by M1‐type macrophages, mostly because M1 macrophages are activated by inflammation and have a significantly higher ability to take up albumin than M0 macrophages.^[^ ^47^, ^48^ ^]^ Next, to further explore the factors that influence the targeting of BMC to M1‐type macrophages, we used different preparations to co‐incubate M1‐type macrophages in parallel. The flow cytometry and quantitative results shown in Figure 3C,D indicate that the uptake of BMC by M1 macrophages was significantly higher than that of BC, indicating that MTX modified on BMC plays an important role in targeting M1 macrophages, which is consistent with our assumption. To further verify the role of FRβ in BMC uptake by cell surface receptors, an FA‐blocking experiment was performed. As shown in Figure 3C,D, the uptake of BMC was obviously inhibited in M1 macrophages pre‐incubated with free FA, further indicating that the pre‐treated FA samples blocked FRβ receptors and suppressed the cellular uptake of BMC., thus confirming that the FRβ‐mediated uptake pathway plays an important role in BMC targeting M1‐type macrophages. The confocal images and semi‐quantitative results also showed consistent results with flow cytometry (Figure S8, Supporting Information). Targeting of BMC to M1 macrophages enables the PDT and PTT effects of CyI and immune cell inhibition of MTX to specifically kill pro‐inflammatory M1 macrophages, alleviate RA inflammation to a greater extent, reduce damage to other cells, and increase the bioavailability of BMC.

The uptake mechanism of BMC by M1 macrophages was also investigated. M1‐type macrophages were pretreated with nystatin, chlorpromazine (CPZ), and colchicine to inhibit caveolin‐dependent, clathrin‐independent, and macropinocytosis. As shown in Figure 3E,F, all inhibitors reduced BMC uptake by M1‐type macrophages. The effects of different inhibitors on BMC uptake by M1‐type macrophages were approximately Nystatin < Colchicine < CPZ, demonstrating that the mechanism by which BMC enters M1‐type macrophages is mainly mediated by clathrin, with a small amount of pinocytosis involved. This is consistent with the previously reported FA targeting of M1‐type macrophage nanoparticles, and the cytoplasmic mechanism of albumin.^[^ ^49^, ^50^ ^]^ Cellular uptake of BMC was evaluated in M1 macrophages using Confocal laser scanning microscope (CLSM) and flow cytometry. As shown in Figure 3G,H, after incubation of BMCs for 6 h, M1‐type macrophages exhibited strong red fluorescence signals, with no significant differences compared to the 8‐h incubation. This suggests that BMC are almost maximally internalized in M1‐type macrophages at 6 h. Next, we verified the above experimental results using flow cytometry, as shown in Figure S9 (Supporting Information), and the results were consistent with the CLSM results, further confirming that BMC could be effectively taken up by M1 macrophages, with saturated uptake at 6 h. Therefore, we chose to apply NIR laser irradiation after incubation for 6 h in subsequent in vitro experiments.

### 2.3 In Vitro Phototherapy of BMC

**Figure 4:**
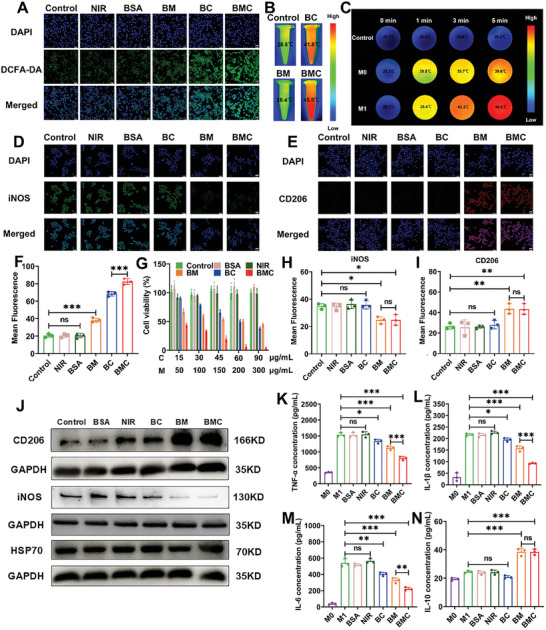
In vitro phototherapy efficacy and anti‐inflammatory action of BMC. (A) Confocal images of intracellular ROS production in M1 macrophages after incubation with different samples; scale bar: 20 µm. (B) Thermal imaging images of M1 macrophages incubated with different samples under NIR irradiation (808 nm, 0.96 W cm^−2^). (C) Thermal images of M0 and M1 macrophages incubated with BMC (CyI: 45 µg mL^−1^) under NIR (808 nm, 0.96 W cm^−2^) irradiation, and M1 macrophages incubated with PBS as the control group. (D) Immunofluorescence survey showing the effects of different sample treatments on the expression of the M1 macrophage marker iNOS; scale bar: 25 µm. (E) Immunofluorescence survey showing the effects of different sample treatments on the expression of the M2 macrophage marker CD206; scale bar: 25 µm. (F) The semi‐quantitative fluorescence analysis of ROS production was performed by “Image J” software. (G) The viability of M1 macrophages was measured by MTT 24 h after various treatments (*n* = 5) (C stands for CyI in BC or BMC, M stands for MTX in BM or BMC). (H) The expression of iNOS in M1 macrophages after treatment with different samples was analyzed by semi‐quantitative fluorescence analysis using “Image J” software. (I) The expression of CD206 in M1 macrophages after different sample treatments was analyzed by semi‐quantitative fluorescence analysis using “Image J” software. (J) Expression of CD206, iNOS, and HSP70 by western blot analysis; (K–N) The secretion of pro‐inflammatory factors (TNF‐α, IL‐1β, and IL‐6) and anti‐inflammatory factors (IL‐10) in macrophages after different treatments was detected using an ELISA kit. MTT data are presented as the mean ± SD, *n* = 5; all other data are presented as the mean ± SD, *n* = 3. *p < 0.05, **p < 0.01, ***p < 0.001, NS: Not significant.

The intracellular ROS level was next evaluated by flow cytometry and confocal laser microscopy using DCFH‐DA as a ROS detector because it produces green fluorescence in the presence of ROS. As shown in **Figure** **4**A,F, compared to the control group, the green fluorescence intensity of the NIR and BSA groups did not show obvious differences, whereas the BM group showed a slight enhancement of the green fluorescence signal, indicating that a trace amount of ROS was produced in the BM group during treatment. This may be because MTX has the effect of nitric oxide decoupling, converting nitric oxide synthase into ROS, resulting in a certain amount of ROS generation.^[^ ^51^, ^52^, ^53^ ^]^ In contrast, the green fluorescence signals of the BC and BMC groups increased, indicating that the CyI‐based nanosystems effectively generated ROS in M1 macrophages after laser irradiation. Between them, BMC exhibited a stronger fluorescence signal than BC, which may be due to MTX increasing intracellular ROS in the process of inhibiting cell proliferation as stated above, as well as the high affinity of MTX for FA receptors on the surface of M1 macrophages, which could increase the uptake of BMC in M1 macrophages, resulting in enhanced ROS generation. These results were further validated by flow cytometry (Figure S10, Supporting Information), which revealed that BMC generated considerably more ROS than the other formulations. These data demonstrate that BMC can produce abundant intracellular ROS to induce apoptosis of M1 macrophages. In addition to RAW264.7 cells, we evaluated the intracellular PDT effect of BMC using THP‐1‐induced polarization of M1 macrophages. As shown in Figure S11 (Supporting Information), the experimental results were consistent with those obtained in RAW 264.7 cells, further confirming the photo‐to‐photodynamic ability of BMC.

The photothermal effects of BMC in vitro on M1 macrophages induced by RAW264.7 and THP‐1 were studied using infrared imaging and thermocouples. As shown in Figure 4B and Figures S12 and S14 (Supporting Information), the experimental results in both cells were generally consistent. Under NIR laser irradiation (808 nm, 0.96 W cm^−2^), the temperature in the CyI‐based BMC‐treated groups of macrophages achieved a thermal jump to a peak of 45.5 °C, whereas the PBS‐ and BM‐treated control groups only showed a slight thermal increase, indicating that BMC possesses good photothermal activity in M1 macrophages. It is worth noting that the BMC group exhibited the strongest photothermal conversion efficiency compared to the BC group, indicating that the high affinity of MTX for FR overexpression in M1 macrophages resulted in enhanced BMC uptake by M1 macrophages. Subsequently, we evaluated the intracellular photothermal effects of different types of macrophages incubated with BMC under NIR irradiation. As shown in Figure 4C and Figure S13 (Supporting Information), in different types of macrophages induced by RAW264.7 cells, the temperature reached up to 45.6 °C in M1 macrophages after laser irradiation for 5 min, whereas the temperature in M0 macrophages only reached up to 39 °C, which cannot achieve the temperature required for thermal ablation. Similarly, as shown in Figure S15 (Supporting Information), in different types of macrophages induced by THP‐1, the temperature of M1 macrophages reached approximately 45 °C after laser irradiation for 5 min, whereas that of M0 and M2 macrophages was less than 40 °C. These results further confirm that BMC can target M1 and protect normal cells and tissues from damage by specifically inducing death of inflammatory cells.

Next, an MTT assay was used to assess whether CyI and MTX have a synergistic killing effect in M1 macrophages. We first evaluated M1 macrophages formed by RAW264.7‐induced polarization. As shown in Figure 4G, laser irradiation alone or BSA alone had almost no killing effect on M1 cells. In contrast, under NIR irradiation, the viability of M1 macrophages treated with BC, BM, or BMC decreased in a dose‐dependent manner. Among them, BMC showed obvious higher inhibitory efficacy than BC and BM. Moreover, at the same concentration of CyI or MTX, BMC exhibited much better killing ability of M1 macrophages than the sum of BC and BM. For instance, when the CyI concentration was 45 µg mL^−1^ and the MTX concentration was 150 µg mL^−1^, BMC showed better killing ability of M1‐type macrophages (83.69% ± 3.41% cell death) than BC (31.97% ± 2.50% cell death) and BM (46.65% ± 2.56% cell death), confirming that the combination of MTX and CyI has a synergistic effect. This may be because MTX, when inhibiting dihydrofolate reductase to reduce dihydrofolate (BH_2_) to tetrahydrofolate (BH_4_), causes nitric oxide synthase uncoupling to increase intracellular ROS levels and increase the sensitivity of immune cells to apoptosis.^[^ ^53^, ^54^ ^]^ To more directly demonstrate the inhibitory effect of BMC on the viability of M1‐type macrophages, we studied the apoptosis and necrosis of M1‐type macrophages by flow cytometry. As shown in Figure S16 (Supporting Information), the percentages of apoptotic and dead cells were as follows: PBS (4.16%) < BSA (4.71%) < NIR (5.09%) < BC (19.25%) < BM (38.26%) < BMC (65.7%). To further verify the aforementioned results, the experiment was repeated in M1 macrophages induced by THP‐1 polarization, and the experimental results (Figures S17 and S18, Supporting Information) were consistent with those in RAW264.7 cells, which further confirmed that BMC could achieve a synergistic effect of photochemotherapy to induce apoptosis in M1 macrophages under NIR irradiation.

### 2.4 In Vitro Polarization of BMC‐Induced Macrophages

The M1 and M2 macrophage polarization types play important roles in the development of RA. Therefore, we next used CLSM to evaluate the in vitro polarization of RAW264.7‐induced macrophages after BMC treatment. Figure 4D,E show in vitro cultures of M1 macrophages after treatment using different preparations, followed by staining for the M1 biomarker (iNOS, green fluorescence) and anti‐inflammatory biomarker (CD206, red fluorescence). As shown, compared to the control groups (control, NIR, BSA, and BC groups), the green fluorescence of macrophages treated with BM or BMC was obviously weakened and the red fluorescence was enhanced, indicating an increase in M2 macrophages, confirming that the MTX‐based nanosystem has the ability to reprogram M1 macrophages into M2 macrophages. The semi‐quantitative analysis and flow cytometry results in Figure 4H,I and Figure S19 (Supporting Information) showed that the fluorescence of FITC anti‐CD206 was significantly enhanced after BM and BMC treatment, indicating that the proportion of M2 macrophages gradually increased, which is consistent with the above experimental results. In addition, the above conclusions were further verified by western blot experiments. As shown in Figure 4J and Figure S20 (Supporting Information), the results are basically consistent with the above conclusions. Similarly, the experiment was repeated in THP‐1‐induced macrophages using flow cytometry. As shown in Figure S21A (Supporting Information), the proportion of M2‐type macrophages increased after BM and BMC treatment, and there is almost no difference between BM and BMC in THP‐1‐induced macrophages repolarization (Figure S21B, Supporting Information), confirming the transformation of M2‐type macrophages after treatment of MTX‐based nanomaterials. These results demonstrate that BMC can specifically kill M1 macrophages after laser irradiation, and that MTX in BMC can further play an immune regulatory role on the viable M1 macrophages, causing them to reprogram into M2 macrophages, achieving a long‐term immune response to alleviate RA inflammation.

It has been reported previously that PTT activates cell protection due to heat stimulation and increases the expression of heat shock proteins such as HSP70.^[^ ^55^, ^56^ ^]^ Studies have confirmed that HSP70 has antioxidant and anti‐inflammatory effects, which can effectively reduce pro‐inflammatory factors.^[^ ^40^ ^]^ Therefore, we evaluated the expression of HSP70 after different treatments by Western blot analysis. As shown in Figure 4J, compared to the control groups (control, NIR, BSA, and BC groups), the expression of HSP70 in BC or BMC group was obviously enhanced, indicating CyI‐based nanomaterials could activate HSP70 expression through PTT.

The levels of pro‐inflammatory factors (TNF‐α, IL‐1β, and IL‐6) and anti‐inflammatory factors (IL‐10) were then detected by enzyme‐linked immunosorbent assay (ELISA) to further confirm the macrophage polarization transition. Compared to RAW264.7 macrophages, macrophages co‐cultured with LPS showed higher levels of TNF‐α, IL‐1β, and IL‐6 (Figure 4L–M). After treatment with PBS, NIR, or BSA, the expression levels of these inflammatory cytokines exhibited a similar tendency to that of M1 macrophages. In contrast, after treatment with BC, BM, or BMC, the secretion of proinflammatory factors decreased, whereas the level of IL‐10 increased (Figure 4N). Among them, BMC exhibited better anti‐inflammatory activity after NIR irradiation. It is worth noting that after BM and BMC treatment, the level of the anti‐inflammatory factor IL‐10 was significantly increased, further suggesting that macrophages are preferentially repolarized from M1‐type to M2‐type after endocytosis of MTX‐based nanosystems. We repeated the above experiment in THP‐1 cells, and the experimental results (Figure S22, Supporting Information) were consistent with the above experimental results, further confirming the experimental conclusions.

### 2.5 In Vivo Targeting of BMC

**Figure 5:**
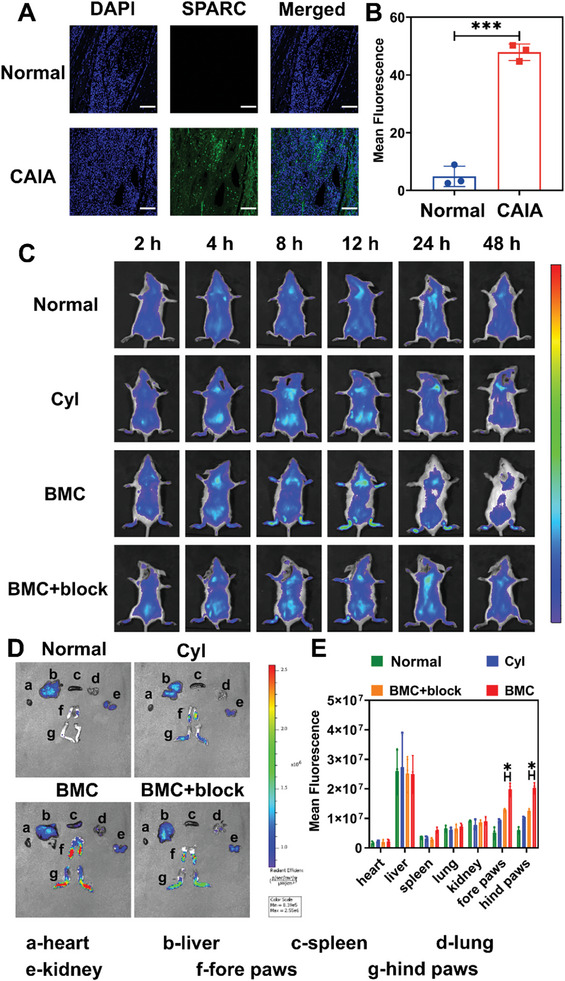
In vivo validation of BMC targeting. (A) The expression of SPARC in synovial tissues of normal and CAIA mice was examined by immunofluorescence staining. (B) The expression of SPARC in (A) was analyzed by fluorescence semi‐quantitative analysis using “Image J” software. All of the data are presented as the mean ± SD, *n* = 3. ***p < 0.001. (C) Fluorescence images of CAIA mice intravenously injected with free CyI or BMC or SPARC blocking with an equivalent dose of BMC at 2, 4, 8, 12, 24, and 48 h. Healthy mice injected with BMC in the tail vein were used as controls. Anti‐SPARC antibody (50 µg) was injected intraperitoneally 1 h in advance to block the binding of BMC and SPARC in CAIA mice. (D) Fluorescence images of the major organs were collected from the different treatment groups. (E) Region of interest (ROI) fluorescence quantification for the organs in Figure 5D. All of the data are presented as the mean ± SD, *n* = 3, *p < 0.05.

To further explore the in vivo targeting properties of BMC in inflammatory joints, a mouse model of collagen antibody‐induced arthritis (CAIA) was established according to a previously reported approach.^[^ ^57^ ^]^ In brief, on day 0, mice were intraperitoneally injected with 4 mg of a 5‐clonal collagen antibody mixture, and on day 3, mice were intraperitoneally injected with LPS (40 µg). On the 7th day, the clinical score of arthritis in mice was observed and recorded, and mice with scores >3 were selected as the CAIA model. Claws were rated on a scale of 0–4 based on the following: 0 = normal claws; 1 = erythema, mild swelling; 2 = mild swelling and erythema from the ankle joint to the tarsal bone; 3 = moderate swelling and erythema, extending from the ankle to the metatarsophalangeal or metacarpophalangeal joints; and 4 = severe edema, joint stiffness, and severe redness of the ankles and feet. After establishing the CAIA model, we first evaluated SPARC protein expression at the inflammation site using immunofluorescence staining, with normal mice used as controls. As shown in **Figure** **5**A,B, the expression level of SPARC protein in normal mice was relatively low, whereas that in the inflammatory site of CAIA mice showed a strong green fluorescence signal, confirming the overexpression of SPARC protein, which is consistent with the results reported in previous literature. As arthritic joints are known to metabolize higher amounts of albumin than healthy tissues, we then studied the targeting properties of BMC to the RA inflammatory site. CyI or BMC was injected into CAIA mice, and the fluorescence distribution of mice at different time points was observed using the IVIS spectral imaging system. As shown in Figure 5C, the fluorescence signal of BMC‐treated mice at the inflammation site (both fore and hind paws) was stronger than that of the CyI or normal groups at all time points, and the maximum fluorescence intensity was reached at 12 h. The images of the isolated organ and claw shown in Figure 5D,E confirmed high BMC accumulation at the site of inflammation. It should be noted that in CyI‐treated mice, the fluorescence intensity at the inflammation sites was higher than that in normal mice, which may have been caused by the destruction of the blood joint barrier at the inflammation site, resulting in vascular leakage. These results revealed that BMC accumulation was higher at the inflammation site in the joints compared to free drugs. To verify that the high accumulation of BMC in the inflammatory tissues of mice is related to the high affinity of SPARC for BMC, we set up SPARC‐blocking CAIA mice to receive equal doses of BMC for fluorescence imaging. As shown, the fluorescence intensity of the inflammatory site in CAIA mice blocked by SPARC was significantly lower than that of the unblocked group at all time points, confirming that the inflammatory site in BMC‐targeted mice was closely related to the high affinity of SPARC for BMC. The above results indicate that the targeting effect of BMC on the inflammatory sites of CAIA mice is due to the nanoscale characteristics of BMC, which can be passively targeted to the inflammatory site through the EVILIS effect, and that the overexpressed SPARC protein in the inflammatory joint has a high affinity for albumin‐based nanosystems.

### 2.6 In Vivo Therapeutic Efficacy of BMC

**Figure 6:**
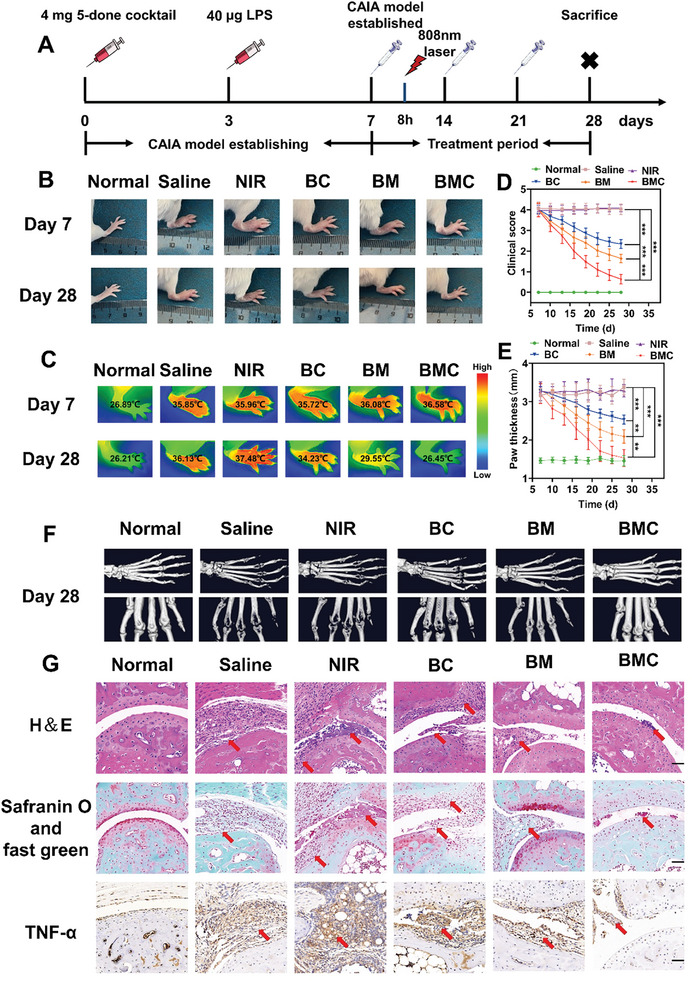
Evaluation of the therapeutic efficacy of BMC. (A) Overall experimental timeline of the in vivo experiment. (B) Representative hind paw images before and after different sample treatments. Top to bottom: hind paw images before treatment (day 7) and after treatment (day 28). (C) Representative hind paw thermal imaging images before and after different sample treatments. (D) Line chart of the mean clinical scores of mice in each treatment group over time during treatment. (E) Line chart of the average thickness of the hind paws of mice in each treatment group over time during treatment. (F) Representative micro‐computed tomography images of the hind paws of CAIA mice in different groups. (G) Histological analysis with hematoxylin and eosin, immunohistochemistry of TNF‐α and safranin‐O, and fast green staining of mice joints in different treatment groups. Scale bar: 50 µm. All data are presented as the mean ± SD; *n* = 5; **p < 0.01, ***p < 0.001.

The treatment plan is illustrated in **Figure** **6**A. CAIA mice were randomly divided into five groups (*n* = 5 per group) and treated with saline (control group), NIR laser only (control group), BM (with MTX dose of 3 mg kg^−1^), BC (with CyI dose of 1 mg kg^−1^) + NIR irradiation (808 nm, 0.96 W cm^−2^), or BMC (with CyI dose of 1 mg kg^−1^, MTX dose of 3 mg kg^−1^) + NIR irradiation (808 nm, 0.96 W cm^−2^). Normal mice were used as controls. Eight hours after tail vein injection, the BC and BMC groups were irradiated with an NIR laser for 5 min. All of the groups were treated every 7 days for three cycles. We took representative photographs of the hind paws of different groups before and after treatment and recorded their clinical scores and toe thickness as indicators of joint inflammation severity and progression. As shown in Figure 6B, severe redness, deformity, and stiffness were observed in the hind paws treated with saline or NIR irradiation alone. The thickness of the hind paws was not significantly changed in the normal group. After treatment with BC or BM, the swelling of the hind paws of mice was relieved significantly, but erythema and stiffness remained. In contrast, mice treated with BMC displayed significant remission of paw swelling and exhibited less redness after 21 days of treatment. In addition, CAIA mice showed significant improvements in clinical scores and a reduction in hind paw thickness after treatment with BMC (Figure 6D,E).

As joint swelling in mice often leads to an increase in the hind paw temperature, we recorded the changes in the hind paw temperature of mice before and after treatment using a thermal imaging camera. As shown in Figure 6C, due to redness and swelling caused by inflammation, the hind paw temperature of mice in the saline and NIR groups increased by approximately 8.96–11.27 °C compared to the normal group. In contrast, after BC, BM, or BMC treatment, the mice experienced mild or moderate relief in the hind paws and their temperature decreased to varying degrees, with the BMC group showing the most pronounced temperature decrease. This indicates that BMC treatment can effectively alleviate the inflammation in the hind paws of CAIA mice.

According to previous research results of our research group, CyI exerts PDT effect when irradiated by NIR, converting oxygen around tissues into cytotoxic ROS.^[^ ^30^, ^31^, ^32^, ^33^, ^34^ ^]^ ROS acts as an inflammatory mediator and may exacerbate inflammation. Hence, in this study, we conducted a dual‐targeting biomimetic nanomaterials for synergistic phototherapy and chemotherapy of RA. MTX was introduced into the nanomaterials to repolarize residue proinflammatory M1 macrophages to anti‐inflammatory M2 macrophages after phototherapy. To confirm the hypothesis, ROS levels in the inflammatory paw of each group were evaluated after treatment on Day 28. As shown in Figure S23 (Supporting Information), compared with the saline group, ROS in the inflammatory paw in BC and BM group showed a decreasing trend, and the decrease of ROS in BMC was much more obviously. This may be because BMC was enriched in the inflammatory site of RA due to the in vivo targeting effect of BSA. After NIR irradiation, CyI in BMC killed pro‐inflammatory M1 cells with high ROS level via PDT and PTT, inhibiting the fountainhead of the inflammation, and MTX further alleviated the inflammation of the paw in the mice. These results further illustrated the potential of chemo‐phototherapy in the treatment of RA.

To determine the erosion of the mice hind paw bone, micro‐CT was performed, and the results are shown in Figure 6F. Significant bone loss and a rough bone surface of the toe joint were observed in saline‐ and NIR‐treated mice, whereas in BMC‐treated mice, the bone integrity was well maintained in the hind paws. Although the BM group still showed bone loss and surface roughness to a certain extent, a significant delaying effect was observed compared to that in the normal saline group. The BC group showed only a slight delay and did not effectively prevent bone damage in the hind paws of mice. In addition, the bone volume to total volume ratio (BV/TV), trabecular thickness (Tb. Th), and trabecular distance (Tb. Sp) were quantitatively analyzed using micro‐CT images. Quantitative analysis of the micro‐CT data further confirmed that the BMC group outperformed all of the other groups (Figure S24, Supporting Information). These results confirm that BMC exerts a significant protective effect on joints.

To further investigate the therapeutic effects of BMC, a series of histological experiments, including microscopic sections stained with hematoxylin and eosin (H&E), safranin O, and fast green, were conducted following treatment. As shown in Figure 6G, compared to normal mice, the saline and NIR groups showed significant joint lesions, such as the erosion of articular cartilage and the invasion of joint cavities by inflammatory cells (red arrow). Similar results were observed for safranin O and fast green staining with glycosaminoglycans in cartilage. Significant proteoglycan loss was observed in CAIA mice treated with saline and NIR, indicating degradation and damage to articular cartilage. In contrast, the infiltration of inflammatory cells was weakened in the BC group, but the erosion of articular cartilage by inflammatory cells still occurred. In the BM group, the articular cartilage damage was less than that in the BC group, but inflammatory cells could still be seen invading the articular cavity. Impressively, no obvious synovial invasion or cartilage destruction occurred in the BMC group. In conclusion, the prepared dual‐targeting BMC nanosystem could alleviate RA symptoms through synergistic photochemotherapy.

The safety of BMC was also assessed via blood biochemical examination and H&E staining of major organs, including the heart, liver, spleen, lung, and kidney. The H&E images did not show any abnormal or pathological changes (Figure S25, Supporting Information). As shown in Figure S26 (Supporting Information), compared to the control group, the indices, including the mean corpuscular concentration (MCV), mean corpuscular hemoglobin concentration (MCHC), mean platelet volume (MPV), hematocrit (HCT), platelet count (PLT), and red cell volume distribution width (RDW) of the blood of the mice injected with BMC, showed no significant abnormalities. In addition, indicators of liver and kidney function, including aspartate aminotransferase (AST), alanine aminotransferase (ALT), blood urea nitrogen (BUN), and creatinine (CREA), were selected to perform serum biochemical tests on mice, with mice injected with normal saline serving as a control group. As shown in Figure S27 (Supporting Information), there was no significant difference between the control and BMC groups, confirming that BMC did not cause liver or kidney damage. Collectively, these experiments demonstrate that BMC exhibits good blood compatibility, biocompatibility, and safety.

### 2.7 In Vivo Anti‐Inflammatory and Macrophage Repolarization by BMC

**Figure 7:**
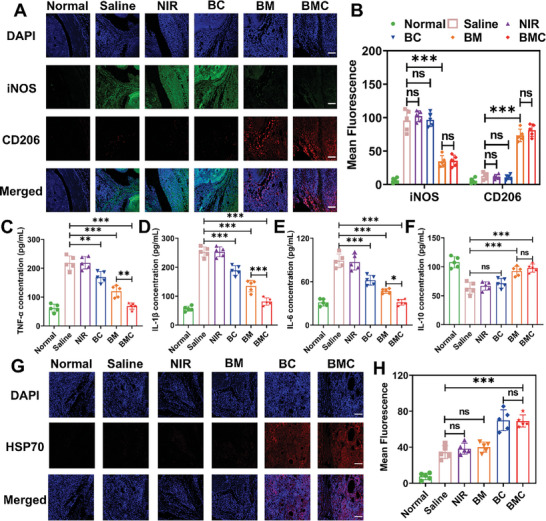
The effects of macrophage polarization and the expression of inflammatory factors were evaluated after treatment. (A) Immunofluorescence analysis of M1 (iNOS, green) and M2 (CD206, red) macrophage markers in the joint after various treatments; scale bar: 100 µm. (B) The expression of iNOS and CD206 in (A) was analyzed by fluorescence semi‐quantitative analysis using “Image J” software. (C–F) The secretion of pro‐inflammatory factors (TNF‐α, IL‐1β, IL‐6) and anti‐inflammatory factor (IL‐10) in the homogenate of the hind paws of mice in different treatment groups was detected using an ELISA kit. (G) Immunofluorescence analysis of HSP70 in CAIA mice; scale bar: 100 µm. (H) Semi‐quantification of fluorescence intensity in Figure 7G determined by “ImageJ” software. All data are presented as the mean ± SD, *n* = 5; *p < 0.05, **p < 0.01, ***p < 0.001, NS: Not significant.

Subsequently, we assessed the polarization of macrophages and inflammatory cytokines in each experimental group exposed to different treatments. As shown in **Figure** **7**A,B, the expression level of the M1 macrophage biomarker iNOS (green) was higher in the saline group, whereas that of the M2 macrophage biomarker CD206 (red) was extremely low, indicating a serious inflammatory response, with a low number of anti‐inflammatory M2 macrophages. The application of irradiation alone did not significantly change this condition. After treating the inflamed joints with BC, there was no conspicuous change in the expression of iNOS and CD206 compared to the saline group, illustrating that although BC treatment has a certain anti‐inflammatory effect, it does not affect the repolarization of macrophages. However, after BM or BMC treatment, iNOS marker expression was significantly decreased, whereas CD206 marker expression was significantly increased. In addition, we also investigated the expression of iNOS and CD206 in mice of all experimental groups by western blot analysis (Figure S28, Supporting Information), and the results were consistent with the above experimental results. These results further confirm the ability of MTX to regulate macrophages in vivo and verify that BMC could reprogram macrophages into anti‐inflammatory M2 macrophages, consistent with the results of the in vitro experiments.

We next evaluated the protein expression level of pro‐inflammatory factor TNF‐α using immunohistochemical staining, as shown in Figure 6G. Compared to the normal group, the saline and NIR groups exhibited significant infiltration of inflammatory cells and the formation of numerous associations, leading to a sharp increase in TNF‐α expression. However, after BC or BM treatment, there was a slight decrease in the number of inflammatory cells and TNF‐α expression. Notably, after BMC treatment, a substantial reduction in inflammatory cell counts was observed, with only minimal tissue adhesion and a significant decrease in TNF‐α expression.

The levels of pro‐inflammatory factors (TNF‐α, IL‐1β, IL‐6) and anti‐inflammatory cytokines (IL‐10) were also investigated using ELISA kits. As depicted in Figure 7C–F, pro‐inflammatory factors remained at high levels in the saline and NIR groups, whereas the IL‐10 levels were lower than those in the normal group. However, after BM or BMC treatment, there was a significant decrease in the levels of pro‐inflammatory factors and a notable increase in the levels of anti‐inflammatory factors, further confirming that the MTX‐based nanosystem reprograms macrophages into anti‐inflammatory M2 macrophages. Notably, after BC treatment, the levels of pro‐inflammatory factors were reduced, but the anti‐inflammatory factors did not show obvious changes compared to the saline group. We hypothesized that the anti‐inflammatory effects of phototherapy are not only due to the specific killing of pro‐inflammatory M1 macrophages but also the activation of HSP70 expression by increasing joint temperature. Therefore, we measured the temperature change in the hind paws of mice during treatment and characterized HSP70 expression. As shown in Figure S29 (Supporting Information), under the irradiation of an NIR laser (808 nm, 0.96 W cm^−2^), the temperature of the BMC group rose to more than 43 °C, meeting the temperature requirements of mild photothermal treatment, indicating the excellent in vivo PTT performance of BMC. Then, we used immunofluorescence staining to investigate the expression of HSP70 after BMC treatment. As shown in Figure 7G,H, the green fluorescence signal was stronger in the BC and BMC groups, whereas the other groups showed no obvious fluorescence signal. This suggests that BMC‐mediated PTT activates HSP70 expression. The above results were further verified by western blot analysis, and as shown in Figure S30 (Supporting Information), the results were consistent with the above experiments. This finding further supports the synergistic abilities of phototherapy and chemotherapy to eliminate M1 macrophages and the ability of MTX to transform M1 macrophages into M2 macrophages. In addition, previous studies have shown that HSP70 and MTX may reduce inflammation by inhibiting the activation of NF‐κB, an inflammatory pathway.^[^ ^58^, ^59^, ^60^ ^]^ It is well known that NF‐κB initiates the activation of the NOD‐like receptor (NLRP) 3‐inflammasome by inducing the expression of NLRP 3. When inflammation occurs, the NLRP inflammasome assembles the NLRP 3 inflammasome, which allows the interaction of the NLRP 3 receptor, Caspase‐1, and ASC aptamer to maintain the inflammatory environment. Therefore, we examined the levels of NF‐κB downstream inflammasome ASC, Casepase‐1, and NLRP3 in the inflammatory paws of mice after different treatments. As shown in Figure S31 (Supporting Information), the levels of ASC, Casepase‐1 and NLRP3 in the paws of mice in BC, BM and BMC groups all decreased, and BMC showed the best inhibitory ability on inflammasome. This may be because the elevation of HSP70 in CyI and MTX inhibits the activation of the NF‐κB pathway and reduces the secretion of downstream inflammasome.

## 3 Conclusion

In this study, we designed an integrated dual‐targeting carrier‐free nanosystem (BMC) for the treatment of RA by combining the hydrophilic targeting ligand BSA, hydrophobic anti‐rheumatic drug MTX, and phototherapeutic reagent CyI using a simple and green self‐assembly method. Due to the overexpression of SPARC protein in the RA joint microenvironment and the inherent high affinity of SPARC for albumin, as well as the structural similarities between MTX and folic acid, BMC could be specifically targeted to the site of RA inflammation and anti‐inflammatory M1 macrophages. In vitro and in vivo experimental results showed that CyI‐mediated phototherapy and MTX‐mediated chemotherapy had a gratifying effect on the synergistic treatment of RA and significantly improved the disease course, demonstrating the potential of the combined application of photo‐chemotherapy in the treatment of RA. Moreover, while synergistically killing anti‐inflammatory M1 macrophages, MTX induced macrophages to reprogram into anti‐inflammatory M2 macrophages, while PTT produced by CyI activated the cells’ own defense mechanism to express the anti‐inflammatory HSP70 protein. Furthermore, the carrier‐free strategy was employed to achieve dual targeting of tissues and cells, effectively avoiding the toxic side effects of phototherapy and chemotherapy and improving the bioavailability of drugs. This study proposes the use of dual‐targeting carrier‐free nanomaterials with photochemotherapy for anti‐inflammatory M1 macrophage apoptosis and as an M2 stimulator in clinical RA treatment and other inflammatory disorders.

## 4 Experimental Section

### Materials

CyI (MW 776.5) was synthesized in the laboratory and bovine serum albumin (BSA) was acquired from Sigma–Aldrich (St. Louis, MO, USA). Methotrexate was purchased from Yuanye Bio‐tech Co. (Shanghai, China). N‐hydroxy succinimide (NHS) and N‐(3‐dimethylaminopropyl) ‐N‐ethyl carbodiimide hydrochloride (EDC) was purchased from Macklin reagent Co. (Shanghai, China). Singlet Oxygen Sensor Green (SOSG) was obtained from Meilun Bio‐tech Co. (Dalian, China). DAPI solution, Methyl thiazolyltetrazolium (MTT), and Annexin V‐FITC/PI apoptosis staining kits were acquired from Solarbio Science &Technology Co. (Beijing, China). The antibodies of SPARC, TNF‐α iNOS, and CD206 were from Affinity Biosciences (Ohio, USA), Protein tech (Wuhan, China), respectively. ELISA test kit of IL‐1β, IL‐10, IL‐6, TNF‐α were purchased from Solarbio Science &Technology Co. (Beijing, China). Immune factors were detected by enzyme label analyzer (Synergy Neo2, Bio Tek, USA).

RAW264.7 cell was obtained from Wuhan Procell Life Science & Technology. Co. Ltd. (Wuhan, China). Cells were cultured in Dulbecco's modified eagle medium (DMEM, Procell, USA) with 10% fetal bovine serum (Procell, USA) and 1% penicillin‐streptomycin (Hyclone, USA). Cells were maintained at standard normoxic culture conditions (21% O_2_, 5% CO_2_, 37 °C). THP‐1 cells were derived from Wuhan Procell Life Science & Technology. Co. Ltd. (Wuhan, China). Cells were cultured in PM1640 with 10% fetal bovine serum (Procell, USA), 0.05 mm β‐mercaptoethanol (Procell, USA), and 1% penicillin‐streptomycin (Hyclone, USA). The cells were maintained under standard atmospheric culture conditions (21% O2, 5% CO2, 37 °C). BALB/c mice (7 weeks old, 15–18 g) were obtained from Beijing Vital River Company (Beijing, China) and kept in 12 h light/12 h dark periods under pathogen‐free conditions with adequate food and water.

### Preparation and Characterization of BSA‐MTX‐CyI (BMC)

Self‐assembled nanomaterials BMC were prepared by coupling carboxyl groups of MTX and CyI with amino groups on BSA. First, MTX (10 mg), EDC (6.83 mg), and NHS (5.06 mg) were dissolved in 1 mL DMSO, and the carboxyl group on MTX was activated by a reaction of 4 h away from light. Similarly, CyI (5 mg), EDC (2.49 mg), and NHS (1.84 mg) were dissolved in 1 mL DMSO, and the carboxyl group on CyI was activated by the reaction without light for 4 h. Next, BSA (5 mg mL^−1^) was dissolved in PBS, and 100 µL MTX solution and 100 µL CyI solution were slowly added to 2 mL BSA solution with intense agitation. After the reaction was completed, a dialysis device with molecular weight of 8000–12 000 kDa was used for purification for 4 days to remove unreacted MTX and CyI. The resulting solution was centrifuged at 12 000 rpm for 5 min to remove the unreacted BSA. The obtained precipitates were washed three times with PBS and then re‐suspended to obtain the completed BMC nanoparticles.

After staining with 5% (w/v) uranium acetate, the size and morphology of the nanoparticles were characterized by transmission electron microscopy (JEOL, JEM‐1200EX), and the hydrated particle size was determined by dynamic light scattering particle size analyzer (NanoZS, Malvern, UK). The contents of MTX and CyI in BMC were determined by UV spectrophotometer at 306 nm and 798 nm, respectively, and calculated by standard curve.

To evaluate the fluorescence imaging capability of BMC, fluorescence emission spectra of CyI and BMC in the range of 700–900 nm were scanned by fluorescence spectrophotometer. To evaluate the serum stability of BMC, BMC were suspended in a high‐glucose medium with DMEM containing 10% FBS at 37 °C for 48 h to simulate the in vivo environment, collected samples, and measured the size change of BMC at different time points. At the same time, the stability of BMC stored in water, PBS and medium (DMEM) at ambient temperature condition for 14 days was determined, and the size of BMC in different media was measured by dynamic light scattering particle size meter (NanoZS, Malvern, UK) every 2 days.

To evaluate the release behavior of MTX, 2 mL of BMC (MTX: 150 µg mL^−1^, CyI: 45 µg mL^−1^) solution was placed in a dialysis box filled with PBS (37 °C) at pH 7.4, and irradiated with or without near‐infrared light (808 nm, 0.96 W cm^−2^) for 5 min. The contents of MTX outside the dialysis bag at different time were detected by ultraviolet spectrophotometer, and the cumulative release rate of drugs was calculated. At the same time, transmission electron microscopy was used to evaluate the release of MTX on BMC at 1, 3, and 5 min.

### Measurement of Photodynamic and Photothermal Effects in Solutions

The formation of ^1^O_2_ was detected by SOSG as an indicator of ^1^O_2_. In simple terms, PBS, BM (MTX:150 µg mL^−1^), BC (CyI: 45 µg mL^−1^) and BMC (MTX:150 µg mL^−1^, CyI:45 µg mL^−1^) were pre‐mixed with a 25 µm SOSG fluorescent probe. Then, the near‐infrared laser (808 nm, 0.96 W cm^−2^) was irradiated for 5 min, and the fluorescence intensity of each sample was measured by enzyme‐labeled instrument (Synergy Neo2, BioTek, USA). (SOSG Ex = 504 nm, Em = 525 nm).

PBS, BM (MTX: 150 µg mL^−1^), BC (CyI: 45 µg mL^−1^), and BMC (MTX: 150 µg mL^−1^, CyI: 1 mL of 45 µg mL^−1^ solution was placed in a 1.5 mL microcentrifuge tube, and the sample was irradiated with a near‐infrared laser (808 nm, 0.96 W cm^−2^) for 5min. Thermal imaging images were recorded with a thermal imager at 0, 1, 3, and 5 min respectively, and the temperature was recorded with a thermocouple thermometer every 30 s.

### In Vitro Study—Cell Polarization Study

The polarization of macrophages was observed by confocal microscope and inverted fluorescence microscope. RAW264.7 cells were inoculated in a 35 mm^2^ confocal culture dish at a density of 1 × 10^5^ cells mL^−1^ and cultured with lipopolysaccharide (LPS, 1 µg mL^−1^) and IL‐4 (50 ng mL^−1^) for 12 h, respectively. THP‐1 cells were stimulated with 200 nm phorbol 12‐myristate 13‐acetate (PMA, Sigma, USA) for 24 h to differentiated to M0 macrophage. The induced M0 macrophages were inoculated in confocal culture dish. Then, the M0 cells were treated with 100 ng mL^−1^ lipopolysaccharides (LPS, Sigma, USA) and 50 ng mL^−1^ interferon‐ γ (IFN‐γ, Sigma, USA) for 24 h to promote M1 polarization, 20 ng mL^−1^ interleukin IL‐4 and 20 ng mL^−1^ IL‐13 (Sigma, USA) for 24 h to promote M2 polarization.

The cells were then fixed with 4% paraformaldehyde. Then the cell morphology was observed with an inverted fluorescence microscope. In addition, for immunofluorescence staining, cells were incubated overnight with iNOS (1:200) or CD206 (1:200) primary antibodies at 4 °C and then incubated with their respective fluorescently labeled secondary antibodies for 30 min. After DAPI staining, the samples were observed by confocal microscopy.

### In Vitro Study—Cell Uptake Study

RAW264.7 cells were inoculated on confocal culture dishes at a density of 1 × 10^5^ cells mL^−1^ and incubated with LPS (1 µg mL^−1^) for 24 h. The original medium was discarded and the dish was added with BMC solution (MTX: 150 µg mL^−1^, CyI: 45 µg mL^−1^), incubated in incubator for 0, 2, 4, 6, and 8 h, fixed cells with 4% paraformaldehyde, washed three times with PBS, and dyed with DAPI dye for 10 min, photographed with confocal laser scanning microscope, and semi‐quantified with Image J software (DAPI Ex = 780 nm, Em = 808 nm).

The efficiency of BMC uptake was evaluated by flow cytometry. RAW264.7 cells were inoculated on six‐well plates at a density of 1 × 10^5^ cells mL^−1^ and incubated with LPS (1 µg mL^−1^) for 24 h. The original medium was discarded, BMC solution (MTX: 150 µg mL^−1^, CyI: 45 µg mL^−1^) was added to the dish, and incubated in the incubator for 0, 2, 4, 6, and 8 h, respectively. After incubation, cells were collected and re‐suspended with 500 µL cold PBS. The fluorescence intensity of each group was determined by CytoFLEX S (Beckman Coulter, USA) and analyzed.

In order to evaluate the cell targeting of BMC, RAW264.7 cells were inoculated in confocal dishes according to the experimental procedures described above and stimulated to differentiate into M1 and M2 macrophages. RAW264.7 cells were M0 macrophages. After cultured for 24 h, the medium was discarded and BMC (MTX: 150 µg mL^−1^, CyI: 45 µg mL^−1^) was added to the dish for incubation for 6 h. The cells were fixed with 4% paraformaldehyde, cleaned with PBS, stained with DAPI, and observed by confocal laser scanning microscope. Semi‐quantitative fluorescence analysis was performed using ImageJ software.

In addition, the targeting of BMC was evaluated using flow cytometry. Similarly, RAW264.7 was inoculated into a six‐well cell culture plate according to the experimental procedure described above, and stimulated to differentiate into M1 and M2 macrophages, while M0 macrophages were not treated. After the culture was completed, the medium was discarded and BMC (MTX: 150 µg mL^−1^, CyI: 45 µg mL^−1^) was added to the dish for incubation for 6 h. CytoFLEX S (Beckman Coulter, USA) was analyzed by flow cytometry after 500 µL cold PBS suspension.

Subsequently, the influencing factors of BMC targeting M1‐type macrophages were evaluated. As previously mentioned, RAW264.7 cells were inoculated in a confocal dish at an appropriate density to stimulate their differentiation into M1‐type macrophages. Subsequently, the medium was removed, BC (CyI: 45 µg mL^−1^) and BMC (MTX: 150 µg mL^−1^, CyI: 45 µg mL^−1^) were added to M1 macrophages for incubation for 6 h, respectively. In addition, folic acid receptor‐blocking experiments were also carried out. To put it simply, cells were pretreated with 1 mm free FA for 1 h to block folate receptors on the surface of M1 cells, and then incubated with BMC (MTX: 150 µg mL^−1^, CyI: 45 µg mL^−1^) for 6 h. Fixation, DAPI staining, confocal microscope observation, and semi‐quantitative analysis using ImageJ software. In addition, flow cytometry was used to evaluate the factors affecting the targeting of M1‐type macrophages by BMC.

Next, the mechanism by which BMC enters the M1 macrophages was investigated. In simple terms, M1 macrophages were treated with chlorpromazine (10 µg mL^−1^), nystatin (15 µg mL^−1^), or colchicine (5 µg mL^−1^) for 1 h under cell culture conditions. The medium was then replaced with serum‐free medium containing BMC (MTX: 150 µg mL^−1^, CyI: 45 µg mL^−1^, 2 mL), incubated for 4 h, and washed with PBS three times. The cells were then fixed with 4% paraformaldehyde. After DAPI staining for 10 min, the samples were observed by confocal microscope. At the same time, after BMC treatment for 4 h, the digestive cells were washed with PBS several times, and PBS was re‐suspended. Fluorescence intensity was collected and analyzed by flow cytometry (CytoFLEX S, Beckman, USA).

### In Vitro Study—In Vitro Phototherapy Efficacy Assay

Intracellular ROS production was detected by DCFH‐DA fluorescent probe. The main steps were as follows: First, RAW246.7 was polarized to M1 macrophages with LPS (1 µg mL^−1^); PBS, PBS+NIR, BSA (3 mg mL^−1^), BM (MTX: 150 µg mL^−1^), BC (CyI: 45 µg mL^−1^) and BMC (MTX: 150 µg mL^−1^, CyI: 45 µg mL^−1^) were added to each group and incubated for 6 h, and the medium was changed after incubation. Fresh medium containing DCFH‐DA (10 µm) was added and incubated for 25 min, and then irradiated with near‐infrared laser (0.96 W cm^−2^, 808 nm) for 5 min. The cells were fixed, cleaned with PBS, and observed with confocal laser microscope. In addition, flow cytometry (CytoFLEX S, Beckman, USA) was used to evaluate ROS production in cells treated with different formulations.

M0‐like macrophages formed by THP‐1 differentiation were stimulated to transform into M1‐like macrophages using LPS+IFN‐γ, and the above experiments were repeated for detection by flow cytometry.

Thermocouple thermometer and thermal imager were used to evaluate the photothermal effect of BMC in different macrophages. RAW264.7 cells were inoculated with 1 × 10^5^ pieces per well into six‐well aseptic cell culture plate, LPS (1 µg mL^−1^) was added to polarize RAW264.7 cells into M1 macrophages, and the untreated RAW264.7 cells were M0 macrophages. The original medium was discarded and incubated with BMC (MTX: 150 µg mL^−1^, CyI: 45 µg mL^−1^) for 6 h, then replaced with fresh medium. M0 macrophages were incubated with PBS for 6 h as the control group. The cells were irradiated with near‐infrared laser (0.96 W cm^−2^, 808 nm), and the temperature was recorded every 30 s. At the same time, the thermal imager was used to record the thermal image of 0, 1, 3, and 5 min.

THP‐1 was inoculated in six‐well aseptic cell culture plate and differentiated into M0 macrophages by adding PMA. M0 macrophages were polarized into M1 or M2 macrophages by adding LPS+IFN‐γ or IL‐3+IL‐14, respectively. BMC (MTX: 150 µg mL^−1^, CyI: 45 µg mL^−1^) was incubated with M0, M1, and M2 macrophages for 6 h, respectively. The cells were irradiated with near‐infrared laser (0.96 W cm^−2^, 808 nm) and the temperature was recorded every 30 s. At the same time, the thermal imager was used to record the thermal image of 0, 1, 3, and 5 min.

Subsequently, the photothermal effects of different preparations in M1 macrophages were evaluated. Consistent with the above experiments, M0 macrophages differentiated from RAW264.7 or THP‐1 were stimulated into M1 macrophages by addition of LPS or LPS+IFN‐γ. The medium was discarded, and PBS, BM (MTX: 150 µg mL^−1^), BC (CyI: 45 µg mL^−1^), and BMC (MTX: 150 µg mL^−1^, CyI: 45 µg mL^−1^) were added to each well and incubated for 6 h, respectively. After centrifugation, the supernant was discarded and the cells were re‐suspended with 200 µL medium. The cells were irradiated with a near‐infrared laser (0.96 W cm^−2^, 808 nm) and the temperature was recorded every 30 s, while thermal images of the highest temperature were taken with a thermal imager.

The cytotoxicity of BMC on M1 macrophages was detected by MTT assay. RAW264.7 was stimulated to M1 macrophages by LPS, and THP‐1‐derived M0 macrophages were stimulated to M1 macrophages by LPS+IFN‐γ. Then the medium was removed and replaced with various samples of different concentrations. After incubation for 6 h, the cells were irradiated with near‐infrared laser (808 nm, 0.96W cm^−2^) for 5 min, and were exchanged into 10 µL MTT solution (5 mg mL^−1^) after 24 h. After 4 h, 110 µL DMSO was added, and the absorbance was detected at 492 nm with a microplate reader (Synergy Neo2, Bio Tek, USA).

Apoptosis was detected using the Annexin V‐FITC apoptosis detection kit according to the manufacturer's protocol. RAW264.7 was stimulated to M1 macrophages by LPS, and THP‐1‐derived M0 macrophages were stimulated to M1 macrophages by LPS+IFN‐γ. The cells were then cultured with different samples (PBS, NIR, BSA, BM, BC, and BMC). Then, near‐infrared light (0.96 W cm^−2^, 808 nm) was irradiated for 5 min. After 24 h, the cells were collected and re‐suspended with 500 µL PBS buffer. Then, add 5 µL FITC‐Annexin and 5 µL PI according to the manufacturer's instructions. Finally, the apoptosis rate was detected by flow cytometry (CytoFLEX S, Beckman, USA).

RAW264.7 was stimulated to M1 macrophages by LPS, and THP‐1‐derived M0 macrophages were stimulated to M1 macrophages by LPS+IFN‐γ, and M0 macrophages were not treated. After induction, M1 macrophages were cultured with different samples (PBS, NIR, BSA, BM, BC, and BMC). Then, near‐infrared light (0.96 W cm^−2^, 808 nm) was irradiated for 5 min. After 24 h of fluid change, the cell supernatant was collected and centrifuged. The obtained supernatant was tested according to the scheme provided in the ELISA kit instructions. After the experiment was completed, the absorbance was measured at 450 nm by the microplate reader (Synergy Neo2, Bio Tek, USA), and the concentration of inflammatory factors in each group was measured according to the standard curve.

### In Vitro Study—Macrophage Phenotype Transition Study

The polarization transformation of macrophages was observed by confocal microscopy and flow cytometry. To determine the M1 and M2 polarization transitions treated with different agents, CD206 was selected as a marker for M2 type macrophages. FITC‐coupled anti‐CD206 antibodies (BD, CA, USA) were used to evaluate macrophage subpopulations. The results were analyzed by flow cytometry (CytoFLEX S, Beckman, USA).

In addition, for immunofluorescence staining, RAW264.7 cells were inoculated into confocal plates and induced into M1 macrophages by LPS (1 µg mL^−1^). After induction, M1 macrophages were combined with PBS, NIR, BSA, BC (CyI: 45 µg mL^−1^) BM (MTX: 150 µg mL^−1^) and BMC (CyI: 45 µg mL^−1^, MTX: 150 µg mL^−1^) were incubated for 6 h, and then irradiated with near‐infrared light (808 nm, 0.96 W cm^−2^) for 5 min. After further incubation for 24 h, the cells were fixed with 4% paraformaldehyde and incubated with iNOS (1:200) or CD206 (1:200) primary antibody at 4 °C overnight. Then they were treated with fluorescently labeled secondary antibodies for 30 min. After staining with DAPI, the samples were observed with confocal microscope (Nikon A1R MP, Japan).

The expression of iNOS and CD206 in M1 macrophages after treatment was detected by western blotting (WB). First, M1 macrophages treated with different formulations were lysed with a cleavage buffer containing protease inhibitors and scraped well with a cell scraper. The protein concentration was then determined by bicinchoninic acid assay (BCA) after centrifugation at 4 °C for 10 min (12 000 rpm). After collecting the supernatant, add 5X loading buffer. The samples were then denatured by boiling at 95 °C for 10 min and separated with 12% (w/v) sodium dodecyl sulfate‐polyacrylamide gel (SDS‐PAGE). The amount of protein contained was 5 µg. The transfer membrane was blocked with 5% skim milk and incubated with primary antibody at 4 °C overnight. Then incubated with enzyme‐labeled secondary antibody for 2 h, the membrane was prepared with ECL substrate.

### In Vivo Study—Establishment of Collagen Antibody‐Induced Arthritis

Balb/c female mice, 8–10 weeks of age, purchased from Beijing Vitonglihua Company (Beijing, China), were reared in pathogen‐free conditions for 12 h light /12 h dark, and given adequate food and water. All animal experiments were conducted at the Animal Center of Qingdao University in accordance with the Animal care guidelines of the National Institutes of Health and the animal control measures of the Ministry of Health of the People's Republic of China. The in vivo study had been approved by the Animal Protection Ethics Committee of Qingdao University. The establishment of CAIA mouse model can be simply summarized as: On day 0, mice were intraperitoneally injected with 4 mg of 5‐clonal collagen antibody mixture; On day 3, mice were intraperitoneally injected with LPS 40µg. On the 7th day, the clinical score of arthritis in mice was observed and recorded, and mice with score >3 were selected as CAIA models. Claws were rated on a scale of 0 to 4 based on the following:0 = normal claws; 1 = erythema, mild swelling; 2 = mild swelling and erythema from ankle joint to tarsal bone; 3 = moderate swelling and erythema, extending from ankle to metatarsophalangeal or metacarpophalangeal joints; 4 = severe edema, joint stiffness, and severe redness of ankles and feet.

### In Vivo Study—The Expression of SPARC Protein was Detected by Immunofluorescence

Synovial tissue was obtained after CAIA mouse model was established. The expression level of SPARC in healthy mice and CAIA mice was detected by immunofluorescence staining. To put it simply, the foot tissues of 3 healthy mice and 3 CAIA mice were taken, and the tissue sections were dewaxed after decalcified embedding section. SPARC (1:200) primary antibody was incubated overnight at 4 °C, and FITC fluorescent‐labeled secondary antibody was incubated for 30 min. The nuclei were stained by DAPI, observed using confocal microscopy, and their fluorescence intensity was semi‐quantitatively analyzed using Image J software.

### In Vivo Study—In Vivo and Ex Vivo Fluorescence Imaging

CAIA mice were induced by the above methods. Mice were intravenously injected with 100 µL free CyI (1 mg kg^−1^) and BMC (CyI: 1 mg kg^−1^), and normal mice were intravenously injected with 100 µL BMC (CyI: 1 mg kg^−1^). In order to further investigate the correlation between the targeting of BMC and the high affinity of SPARC protein overexpressed at the inflammatory sites of BSA and RA, a SPARC blocking experiment was established. In order to block the binding of SPARC and BMC in vivo, 50 µg anti‐SPARC antibody was intraperitoneally injected into CAIA mice 1 h ahead of time, and then 100 µL BMC (BMC:1 mg kg^−1^) was injected into the tail vein of CAIA mice pre‐saturated with SPARC. Fluorescence images were obtained by IVIS imaging system at 2, 4, 6, 8, 12, 24, and 48 h after injection. After 48 h post‐injection, foot and major organs of mice were excised for ex vivo imaging.

### In Vivo Study—In Vivo PTT Assay

In order to evaluate the photothermal effect of BMC in vivo, the mice were administered according to the above treatment methods. After 8 h post‐injection, the mice were irradiated with 808 nm NIR light at 0.96 W cm^−2^ for 5 min, and the temperature changes of the foot paw were carefully recorded with a thermocouple thermometer every 30 s.

### In Vivo Study—In Vivo ROS Assay

ROS remnants within the infected ankle were detected using the Tissue ROS Assay Kit. In brief, 50 mg of fresh ankle cartilage tissue was taken, 1 mL homogenate buffer was added, and fully homogenized by tissue homogenizer. After centrifugation at 4 °C for 10 min, the supernatant was collected in a 96‐well plate. After adding 10 L probe and placing for 30 min in the dark, the OD was measured by a fluorescent microplate reader (BBoxiProbe O13 Ex = 488 nm; Em = 606 nm). Fresh articular tissue from each group was collected and 9 x volume saline homogenate was added to prepare 10% tissue homogenate. Supernatant was collected after centrifugation (3000 rpm, 10 min).

### In Vivo Study—In Vivo Therapeutic Experiments

To evaluate the therapeutic effect of BMC, CAIA mice were randomly divided into 5 groups (*n* = 5). 200 µL of Saline, NIR, BC (CyI: 1 mg kg^−1^), BM (MTX: 3 mg kg^−1^) or BMC (CyI: 1 mg kg^−1^, MTX: 3 mg kg^−1^) was intravenous administration, and normal group was set as control group. The above preparations were administered every 7 days for three times. It should be noted that in order to achieve the best effect of phototherapy, the feet of CAIA mice with were irradiated with laser (0.96 W cm^−2^, 808 nm) for 5 min at 8 h after administration. The mice were evaluated every 3 days after the first injection. Clinical scores were measured according to the scoring criteria. The thickness of the right rear paw (mm) of each mouse was measured with a vernier caliper. After treatment, the mice were killed, and the right hind limb of one mouse in each group was excised. All specimens were then fixed in 10% formalin and decalcified with EDTA solution for 3 weeks. The joint tissues were embedded in paraffin wax, and the paraffin sections were stained with H&E or safranin‐O. The expression of TNF‐α in each group was detected by immunohistochemical staining. In addition, the joint homogenates were prepared from the inflammatory joints of three mice in each group. The concentrations of IL‐10, IL‐1β, IL‐6, and TNF‐α were detected by enzym‐linked immunosorbent assay (ELISA) (Servicebio) according to the steps provided by the manufacturer.

### In Vivo Study—Micro‐CT Scanning

After treatment, CAIA mice in different treatment groups and normal mice were scanned by 360° on the animal micro‐CT scanner (Quantum GX2, PerkinElmer, JP). The scanned claws were reconstructed into a 3D structure by the filtered post‐projection method.

### In Vivo Study—Immunofluorescent Staining

Tissue sections were dewaxed, treated with iNOS, CD206, and HSP70 primary antibodies overnight at 4 °C, and then incubated with FITC fluorescently labeled secondary antibodies or rhodamine fluorescently labeled antibodies for 30 min. After staining the nuclei with DAPI, the sections were imaged with confocal laser scanning microscopy. The expression of iNOS, CD206, and HSP70 in joint supernatant was detected by WB.

### In Vivo Study—Blood Routine Test

Orbital blood was taken from Balb/c mice treated with saline and BMC. The blood obtained was immediately mixed with anticoagulants, and then whole blood was detected by automatic blood cell analyzer.

### In Vivo Study—Liver and Kidney Function Test

Orbital blood was taken from Balb/c mice injected with normal saline and BMC. The obtained blood was centrifuged at 4 °C overnight and treated with supernatant at 3000 rpm for 15 min. Then, two items of liver and kidney function were detected by automatic biochemical analyzer. Including alanine aminotransferase (ALT), aspartate aminotransferase (AST), urea nitrogen (BUN) and blood creatinine (CREA).

### Statistical Data Analysis

All experimental data were exhibited as mean ± standard deviation (SD), and the statistical significance among different groups was analyzed by Student's t test with a value of *p < 0.05, **p < 0.01, and ***p < 0.001.

## Conflict of Interest

The authors declare no conflict of interest.

## Supporting information



Supporting Information

## Data Availability

The data that support the findings of this study are available from the corresponding author upon reasonable request.
