# Supplementary material for: Dual Targeting Biomimetic Carrier‐Free Nanosystems for Photo‐Chemotherapy of Rheumatoid Arthritis via Macrophage Apoptosis and Re‐Polarization
Source: Adv Sci (Weinh). 2025 Jan 22;12(10):2406877. doi: 10.1002/advs.202406877 (PMC11904978; doi:10.1002/advs.202406877)
Supplement: Supplementary file 1 — Supporting Information [file ADVS-12-2406877-s001.docx]

**Supporting Information**

**Dual Targeting Biomimetic Carrier-Free Nanosystems for Photo-Chemotherapy of Rheumatoid Arthritis via Macrophage Apoptosis and Re-polarization**

Guanghe Xue ^a,#^, Huimei Jiang ^a,#^, Zhenhua Song ^b,#^, Yifan Zhao ^a^, Wen Gao ^c^, Bai Lv ^a^, Jie Cao ^a,^*

^a^ Department of Pharmaceutics, School of Pharmacy, Qingdao University, Qingdao, 266071, China.

^b^ Department of Pharmacology, School of Pharmacy, Qingdao University, Qingdao, 266071, China.

^c^ Department of Radiation Oncology, The Affiliated Hospital of Qingdao University, Qingdao, 266000, China

# These authors contributed equally to this manuscript

* Corresponding email: [caojie0829@qdu.edu.cn](mailto:caojie0829@qdu.edu.cn)

**Supporting Figures**





**Figure S1.** Hydrated particle size change of BMC in different media at ambient temperature condition for 14 days.





**Figure S2.** Change of hydrated particle size of BMC in 10% FBS+DMEM medium for 48 h at 37℃.


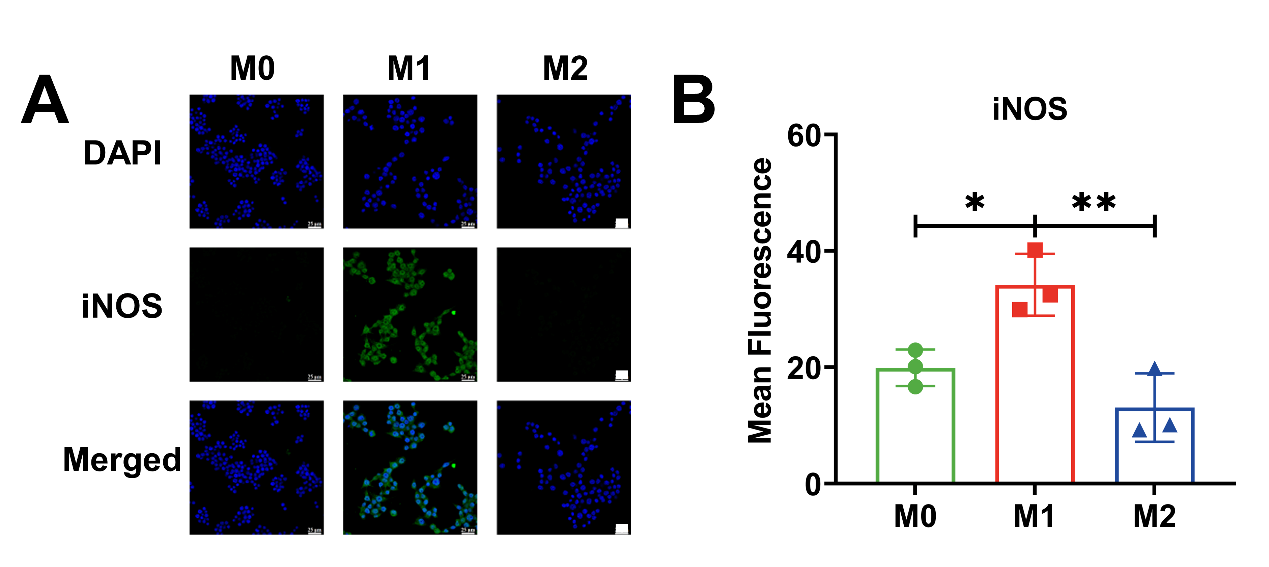


**Figure S3.** (A) Confocal images of the expression of M1 macrophage marker iNOS in different cells (RAW 264.7), Scale bar is 25 μm. (B) Semi quantitative fluorescence analysis of iNOS expression in different cells evaluated by "Image J" software (n=3, *P<0.05, **P<0.01).


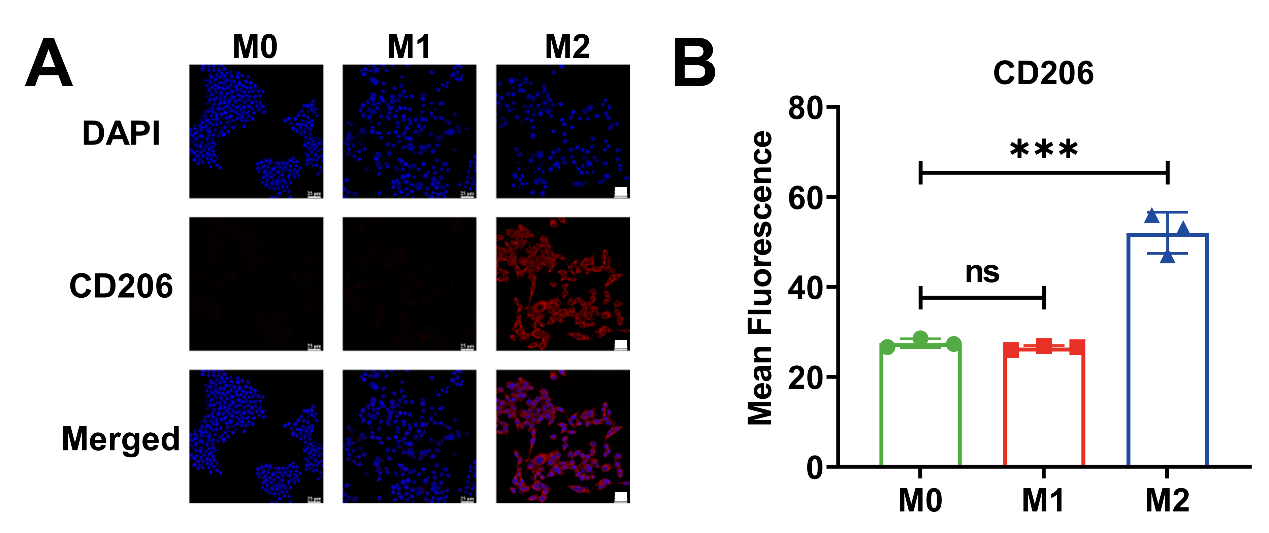


**Figure S4.** (A) Confocal images of the expression of M2 macrophage marker CD206 in different cells (RAW 264.7), Scale bar is 25 μm. (B) Semi quantitative fluorescence analysis of CD206 expression in different cells evaluated by "Image J" software (n=3, NS: Not Significant, ***P<0.001).


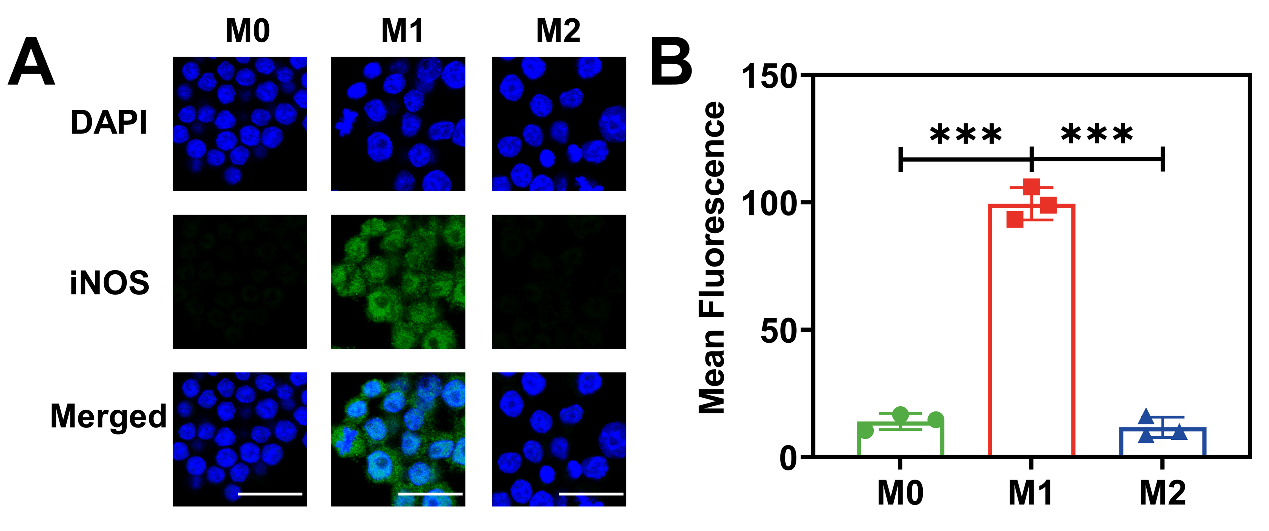


**Figure S5.** (A) Confocal images of the expression of M1 macrophage marker iNOS in different cells (THP-1), Scale bar is 25 μm. (B) Semi quantitative fluorescence analysis of iNOS expression in different cells evaluated by "Image J" software (n=3, ***P<0.001).


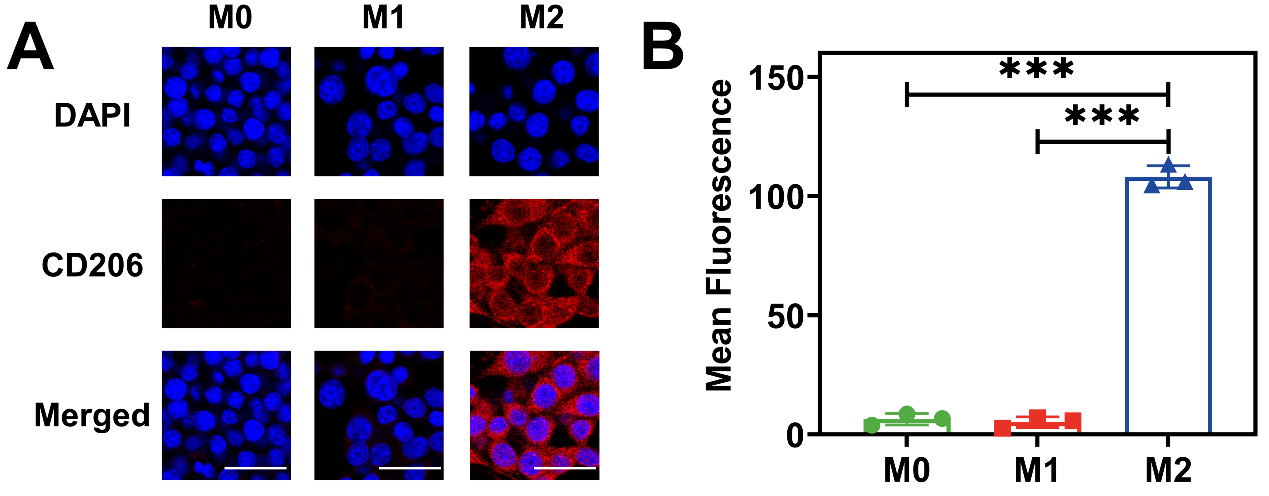


**Figure S6.** (A) Confocal images of the expression of M2 macrophage marker CD206 in different cells (THP-1), Scale bar is 25 μm. (B) Semi quantitative fluorescence analysis of CD206 expression in different cells evaluated by "Image J" software (n=3, ***P<0.001).


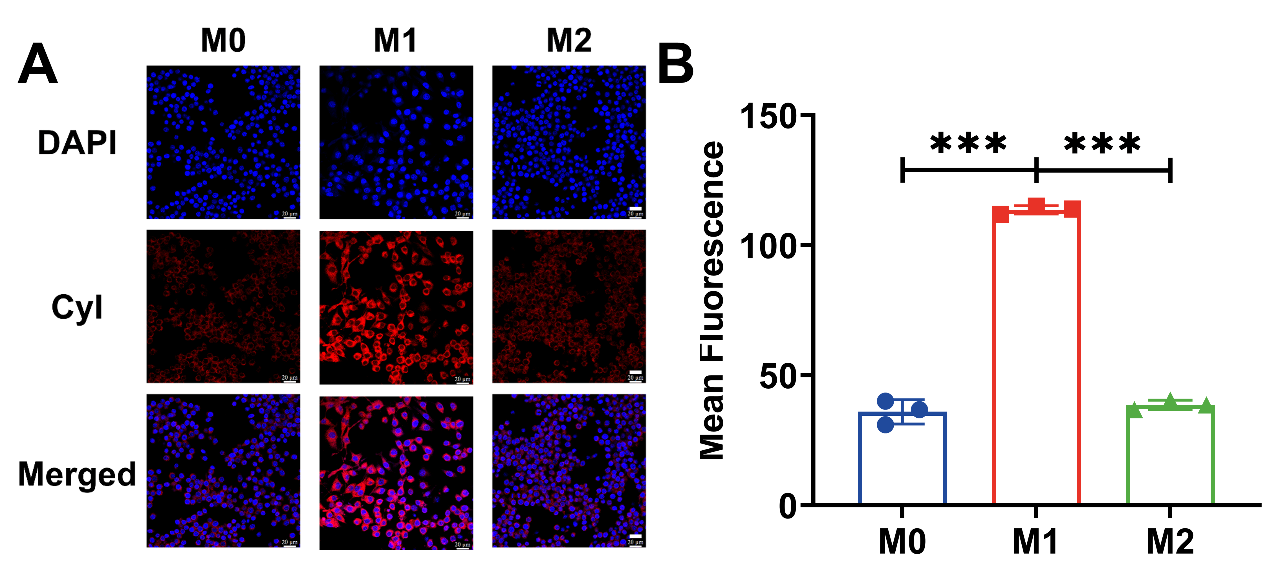


**Figure S7.** (A) Confocal fluorescence images of BMC (CyI: 45 μg/ml) incubated with different types of macrophages (RAW 264.7), scale bar is 20 μm. (B) The results in Figure (A) were analyzed by fluorescence semi-quantitative analysis using Image J software (n=3, ***P<0.001).


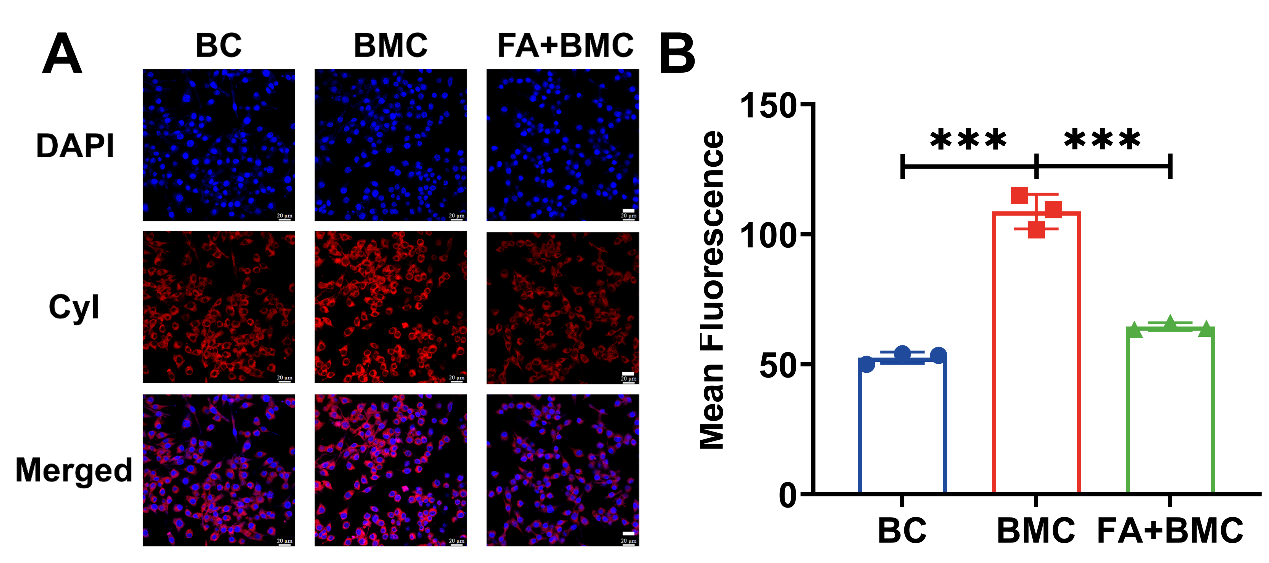


**Figure S8.** (A) Confocal fluorescence images of M1 macrophages (RAW 264.7) incubated with PBS, BC, BMC and FA+BMC (CyI content 45 μg/ml) for 6 h, scale bar is 20 μm. (B) The results in Figure (A) were analyzed by fluorescence semi-quantitative analysis using Image J software (n=3, ***P<0.001).


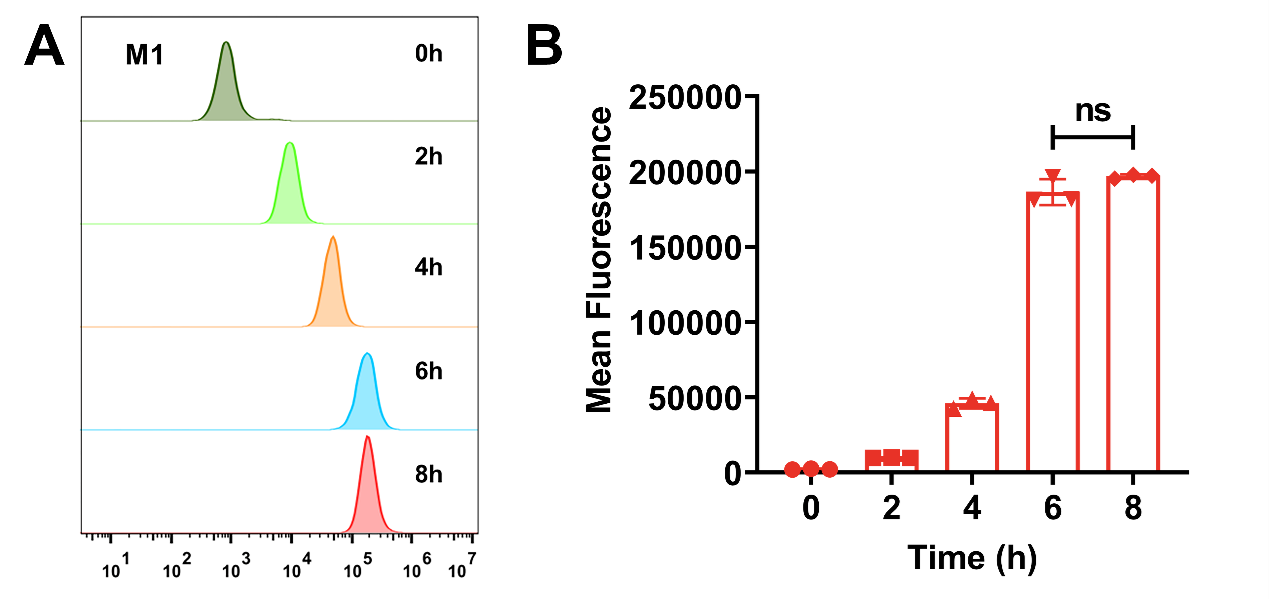


**Figure S9.** (A) The efficiency of BMC uptake by M1 macrophages (RAW 264.7) at different times was analyzed by flow cytometry. (B) Flowjo software was used for fluorescence semi-quantitative analysis of the results (n=3, NS: Not Significant).


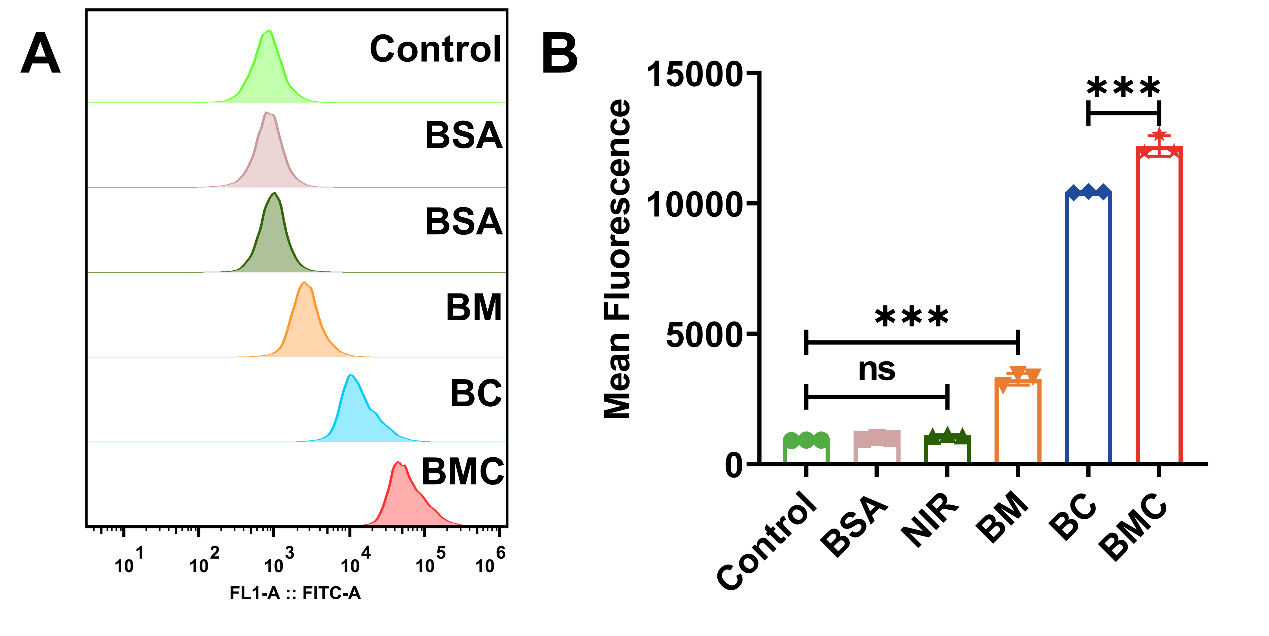


**Figure S10.** (A) The ROS production was analyzed by flow cytometry after M1 macrophages (RAW 264.7) incubated with different preparations for 6 h and irradiated with NIR (0.96 W/cm^2^, 808 nm) for 5 min. (B) The results in Figure S10A were analyzed by fluorescence semi-quantitative analysis using Flowjo software (n=3, NS: Not Significant, ***P<0.001).


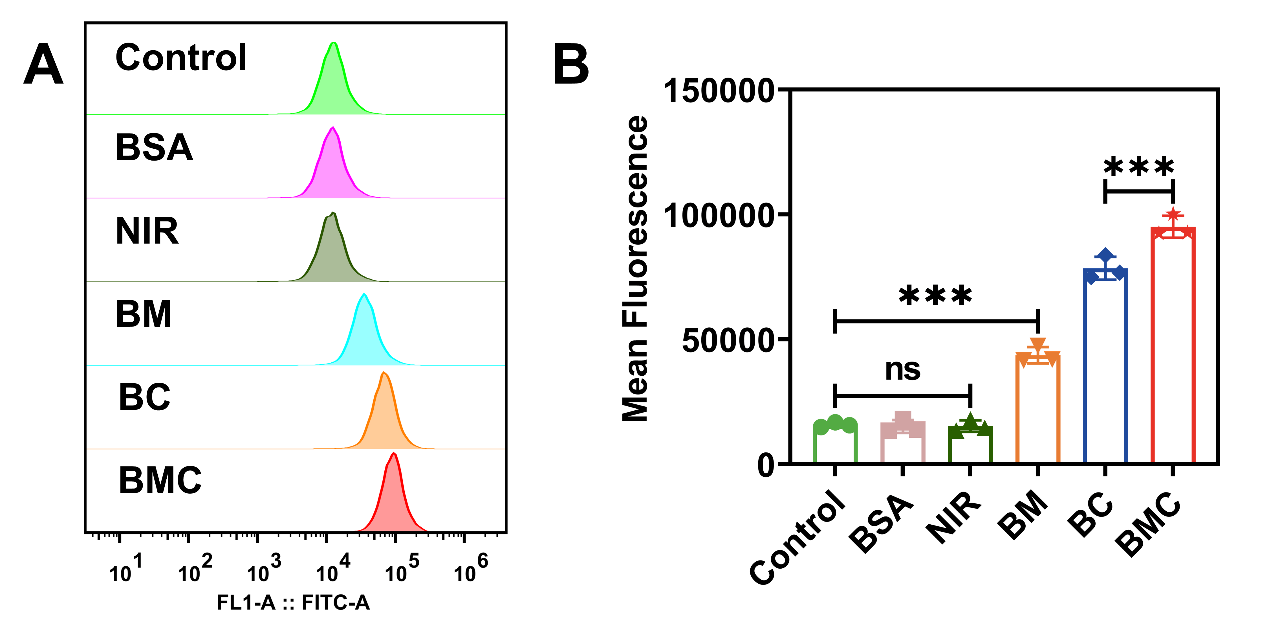


**Figure S11.** (A) The ROS production was analyzed by flow cytometry after M1 macrophages (THP-1) incubated with different preparations for 6 h and irradiated with NIR (0.96 W/cm^2^, 808 nm) for 5 min. (B) The results in Figure S11A were analyzed by fluorescence semi-quantitative analysis using Flowjo software (n=3, NS: Not Significant, ***P<0.001).





**Figure S12.** Temperature change curves of M1 macrophages (RAW 264.7) incubated with different samples under NIR (808 nm, 0.96 W/cm^2^) irradiation (n=3).





**Figure S13.** Temperature variation curve of M0 and M1 macrophages (RAW 264.7) incubated by BMC (CyI: 45 μg/mL) under NIR (808 nm, 0.96 W/cm^2^) irradiation, and M1 macrophages incubated with PBS as the control group (n=3).


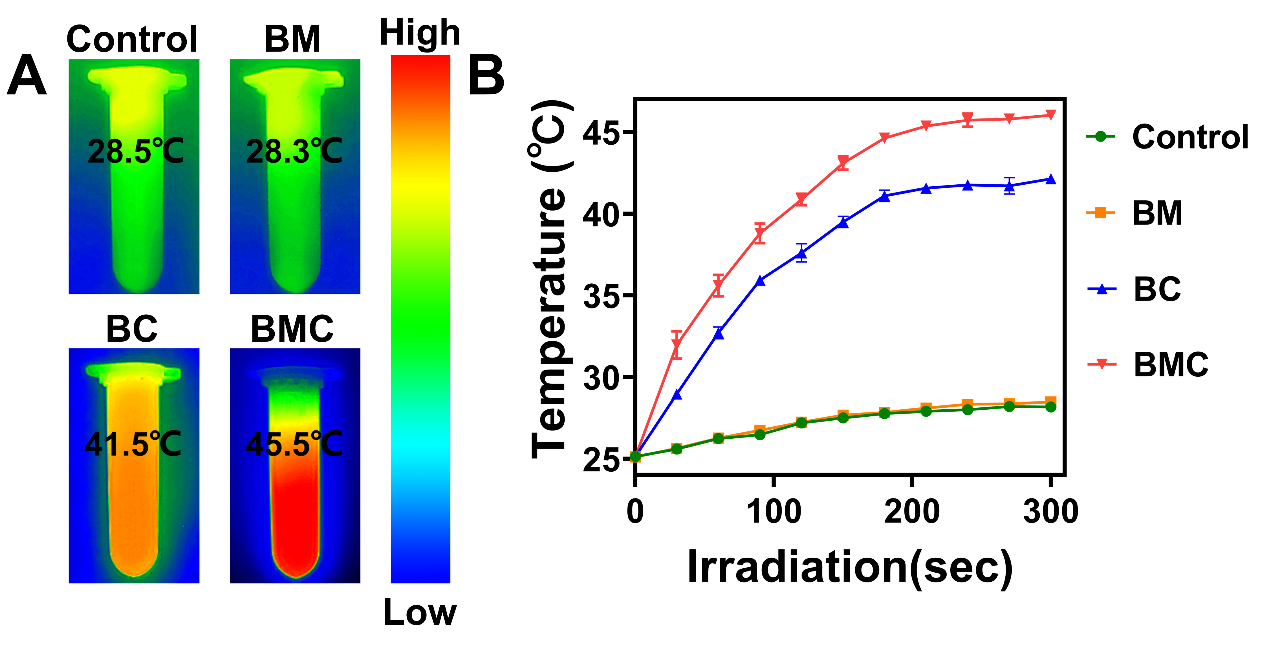


**Figure S14.** (A) Thermal imaging images of M1 macrophages (THP-1) incubated with different samples under NIR irradiation (808 nm, 0.96 W/cm^2^). (B) Temperature change curves of M1 macrophages (THP-1) incubated with different samples under NIR (808 nm, 0.96 W/cm^2^) irradiation (n=3).


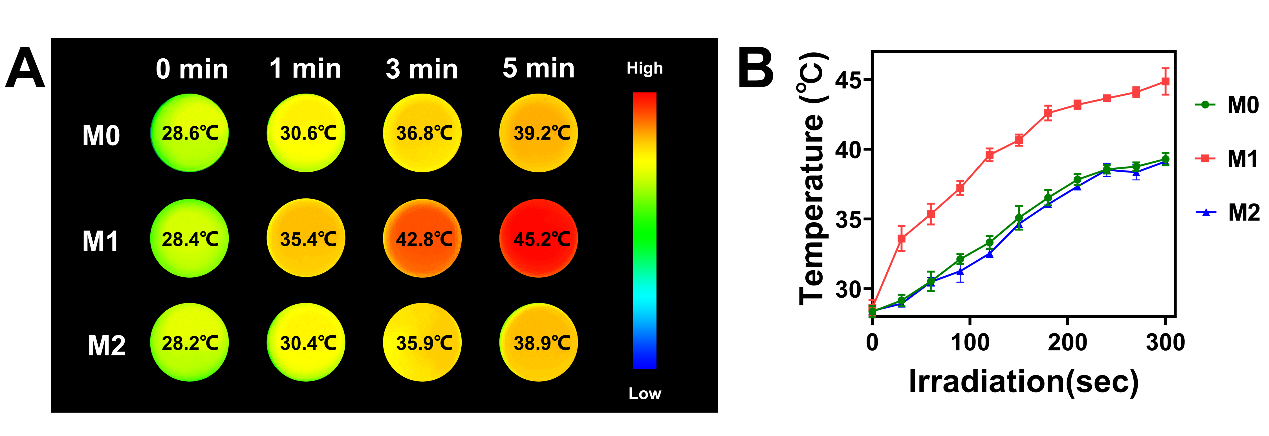


**Figure S15.** (A) Thermal images of M0, M1and M2 macrophages (THP-1) incubated with BMC (CyI: 45 μg/mL) under NIR (808 nm, 0.96 W/cm^2^) irradiation. (B) Temperature change curves of M0, M1 and M2 macrophages (THP-1) incubated with BMC (CyI: 45 μg/mL) under NIR (808 nm, 0.96 W/cm^2^) irradiation (n=3).


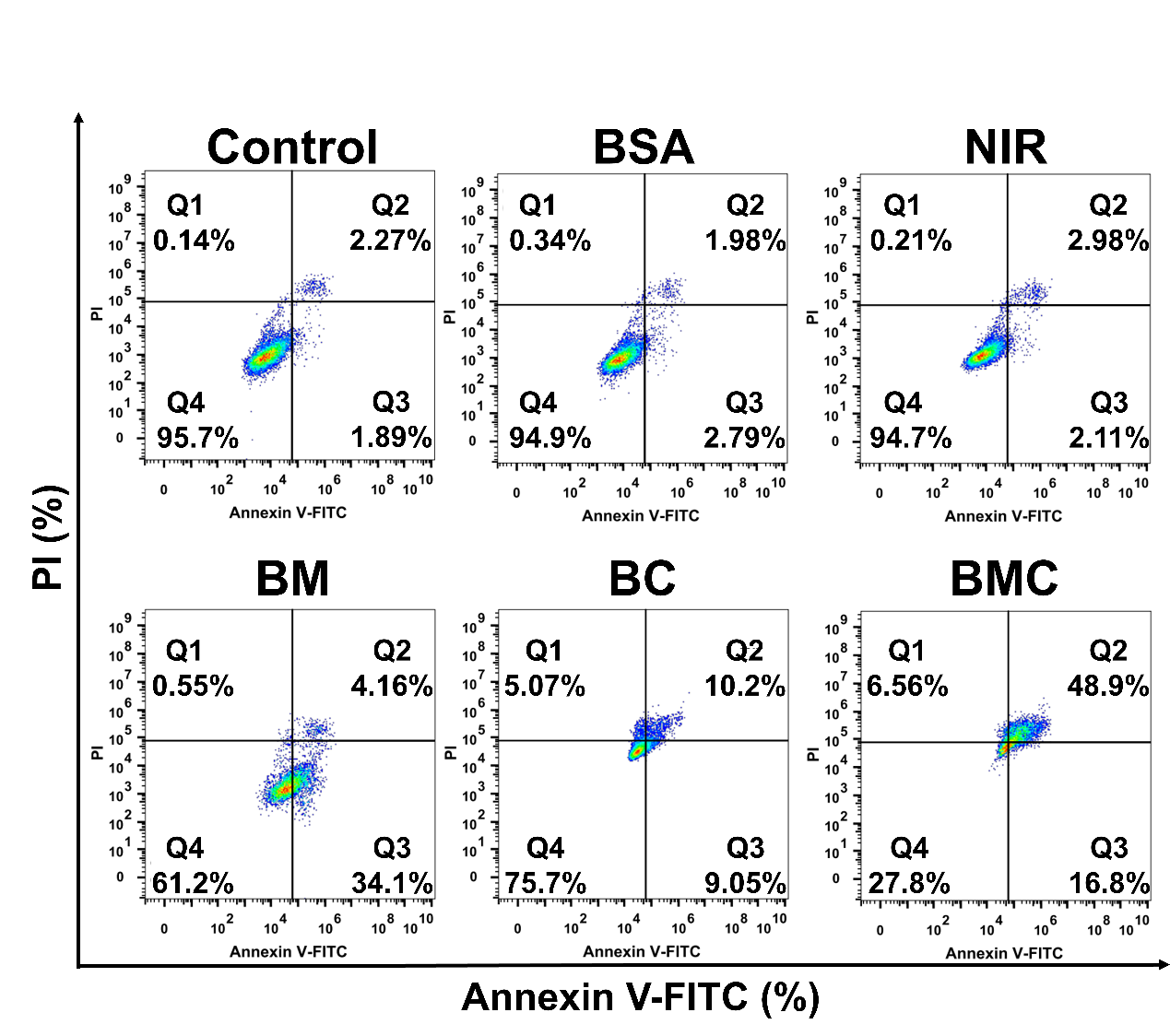


**Figure S16.** Flow cytometry analysis of apoptosis of the M1-type macrophages (RAW 264.7) quantified by Annexin V biomarker intensity after administration of various preparation treatments.


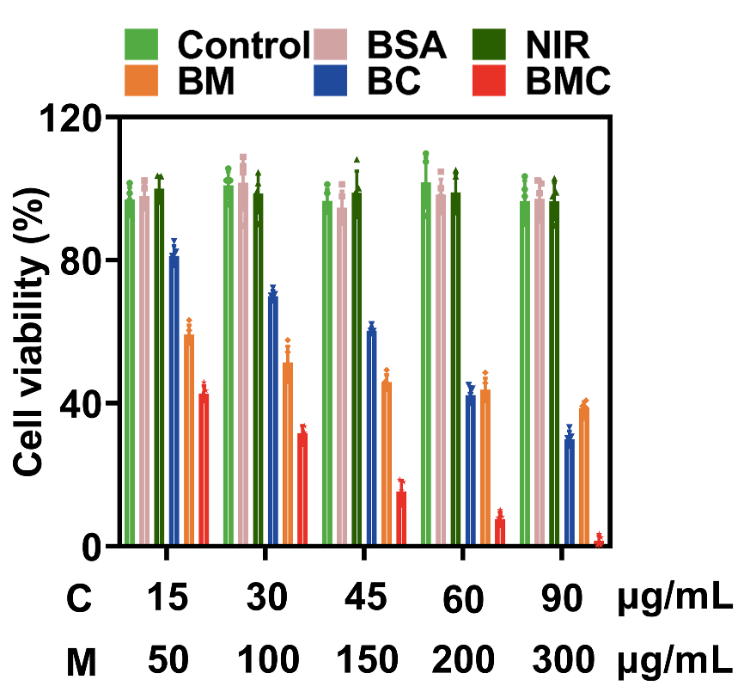


**Figure S17.** The cell viability of M1 macrophages (THP-1) was measured by MTT 24 h after various treatments (n = 5) (C stands for CyI in BC or BMC, M stands for MTX in BM or BMC).


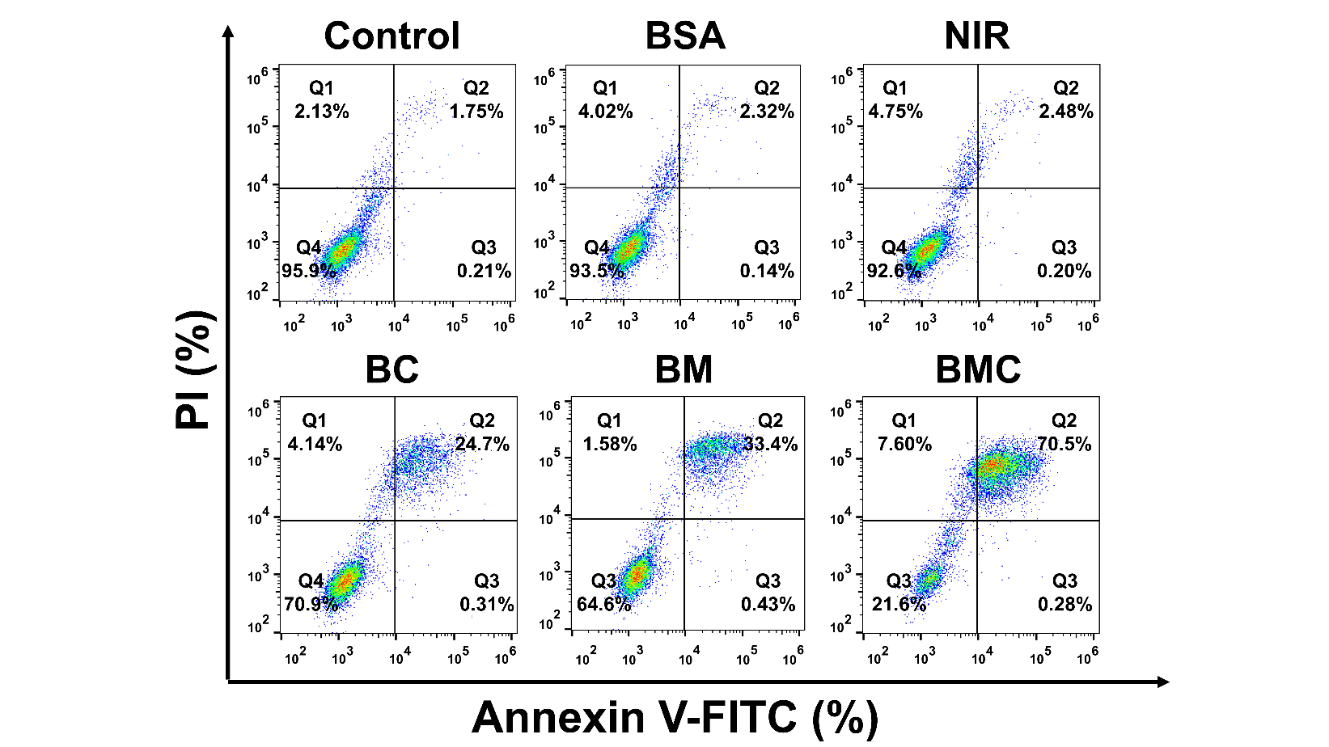


**Figure S18.** Flow cytometry analysis of apoptosis of the M1-type macrophages (THP-1) quantified by Annexin V biomarker intensity after administration of various preparation treatments.


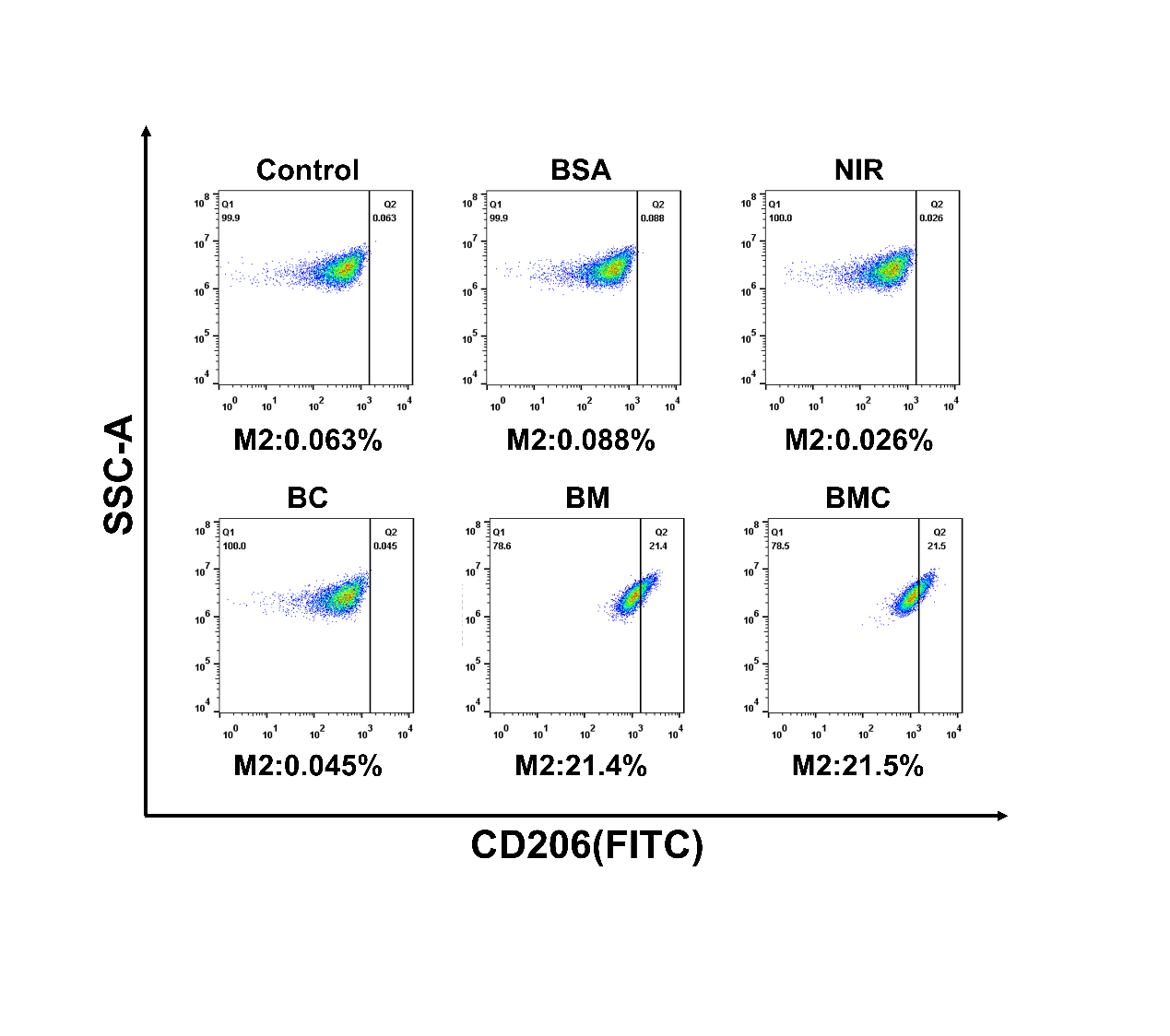


**Figure S19.** Flow cytometry was used to determine the percentage of M2 (CD206-positive) macrophages in M1 macrophages (RAW 264.7) treated with different formulations.

**
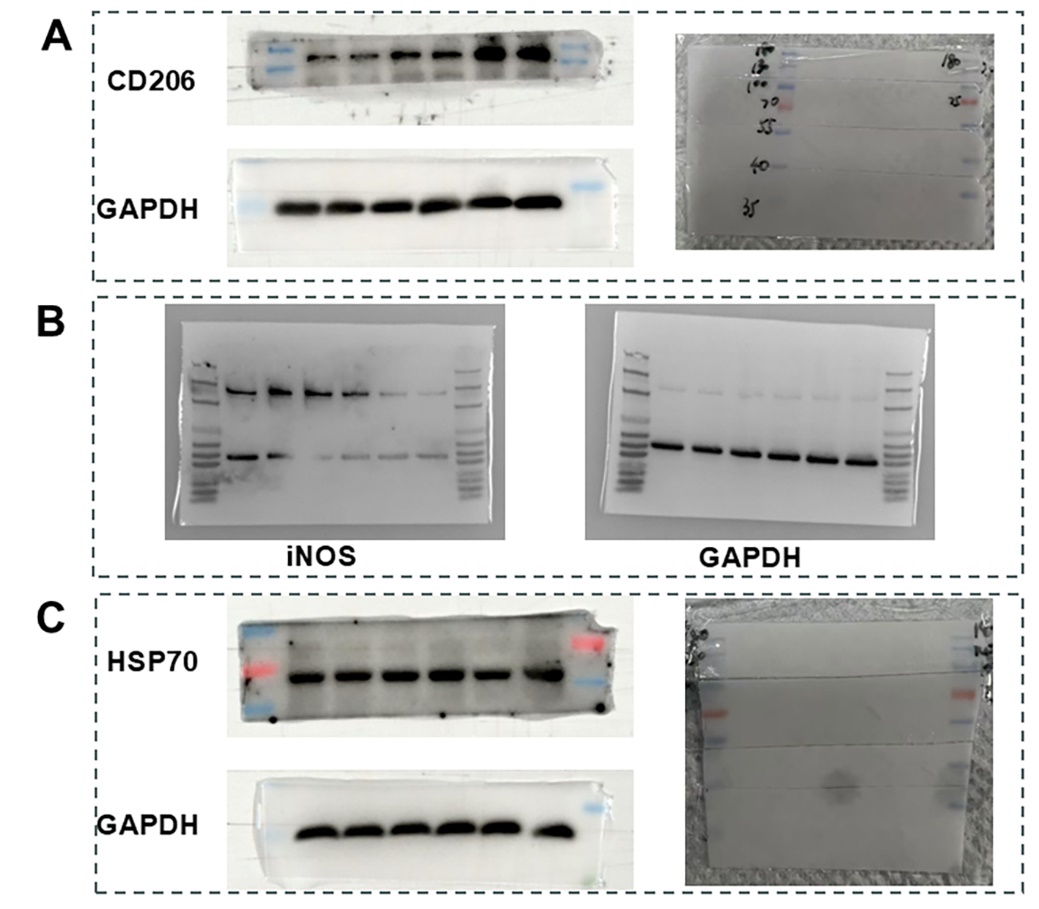
**

**Figure S20.** Images of (A) CD206, (B) iNOS, and (C) HSP70 by western blot.

*
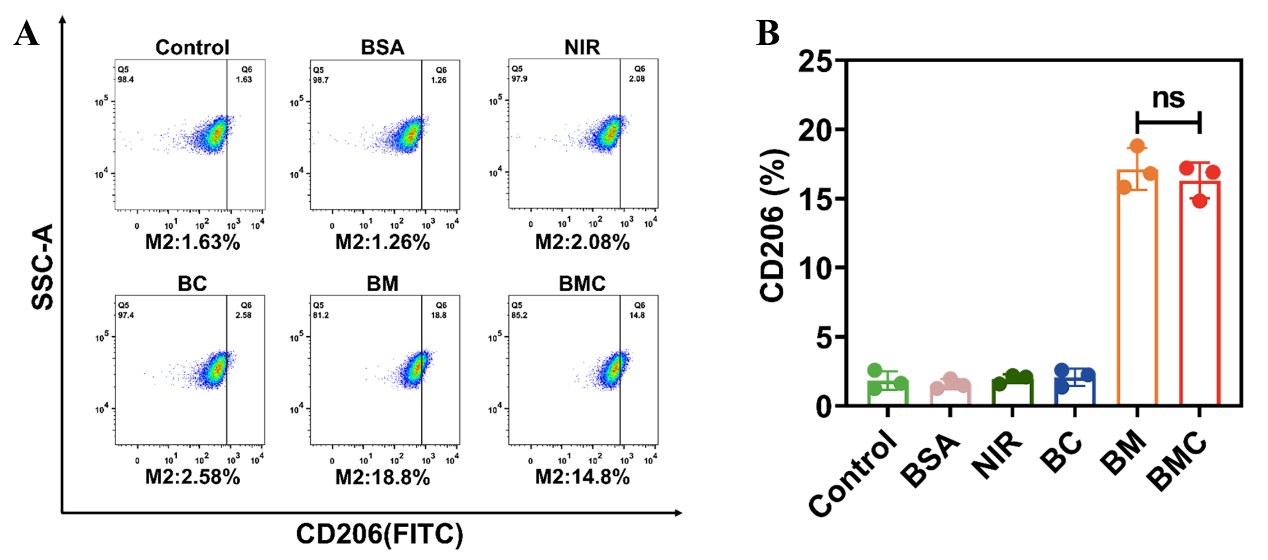
*

**Figure S21.** (A) Flow cytometry was used to determine the percentage of M2 (CD206-positive) macrophages in M1 macrophages (THP-1) treated with different formulations. (B) Statical analysis of the percentage of M2 macrophages in M1 macrophages (THP-1) treated with different formulations. All data are mean ±SD, n=3, NS: Not Significant.


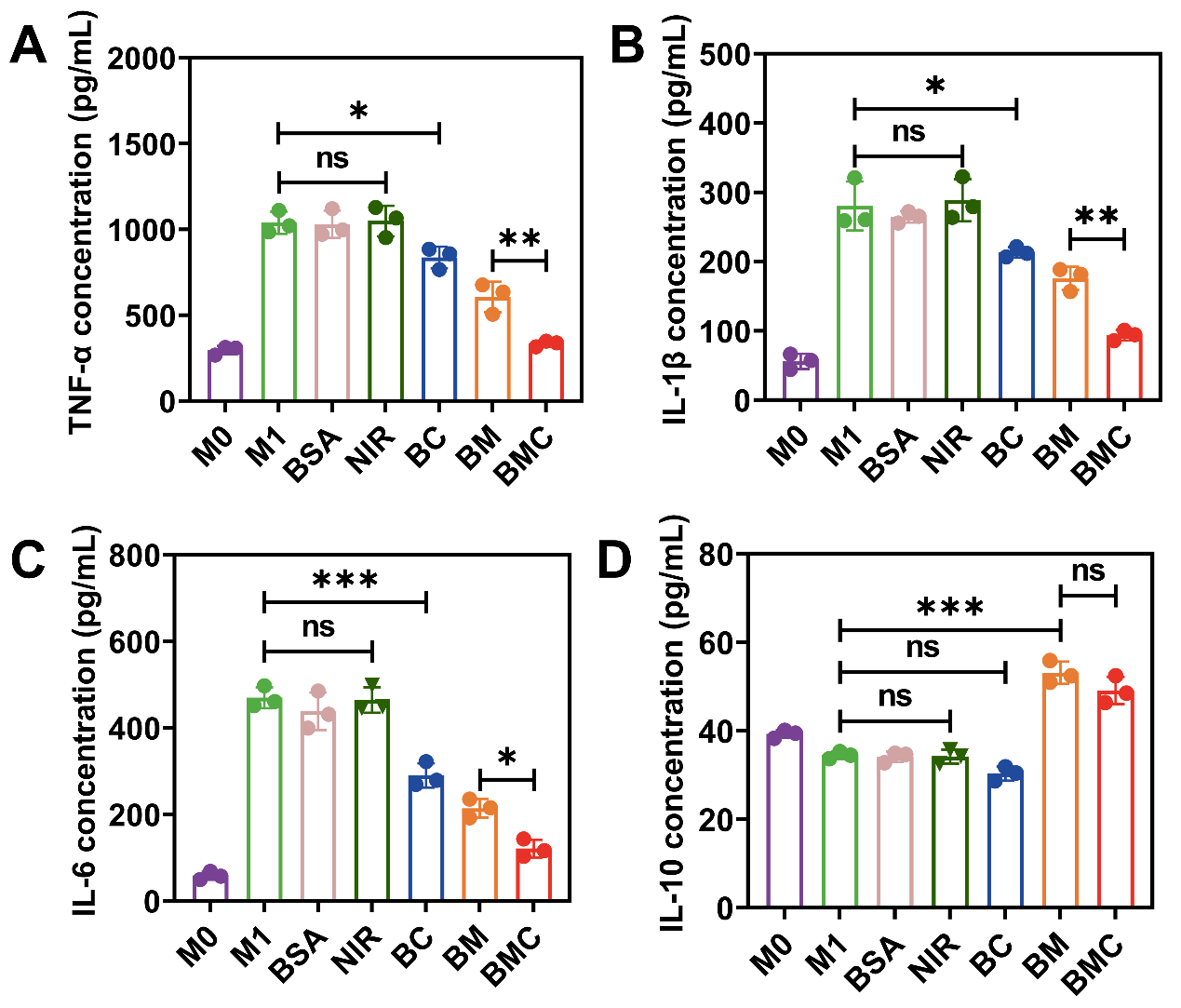


**Figure S22.** (A-D) The secretion of pro-inflammatory factors (TNF-α, IL-1β and IL-6) and anti-inflammatory factors (IL-10) in M1 macrophages (THP-1) were detected by ELISA kit (n=3, *P<0.05, **P<0.01, ***P<0.001, NS: Not Significant).


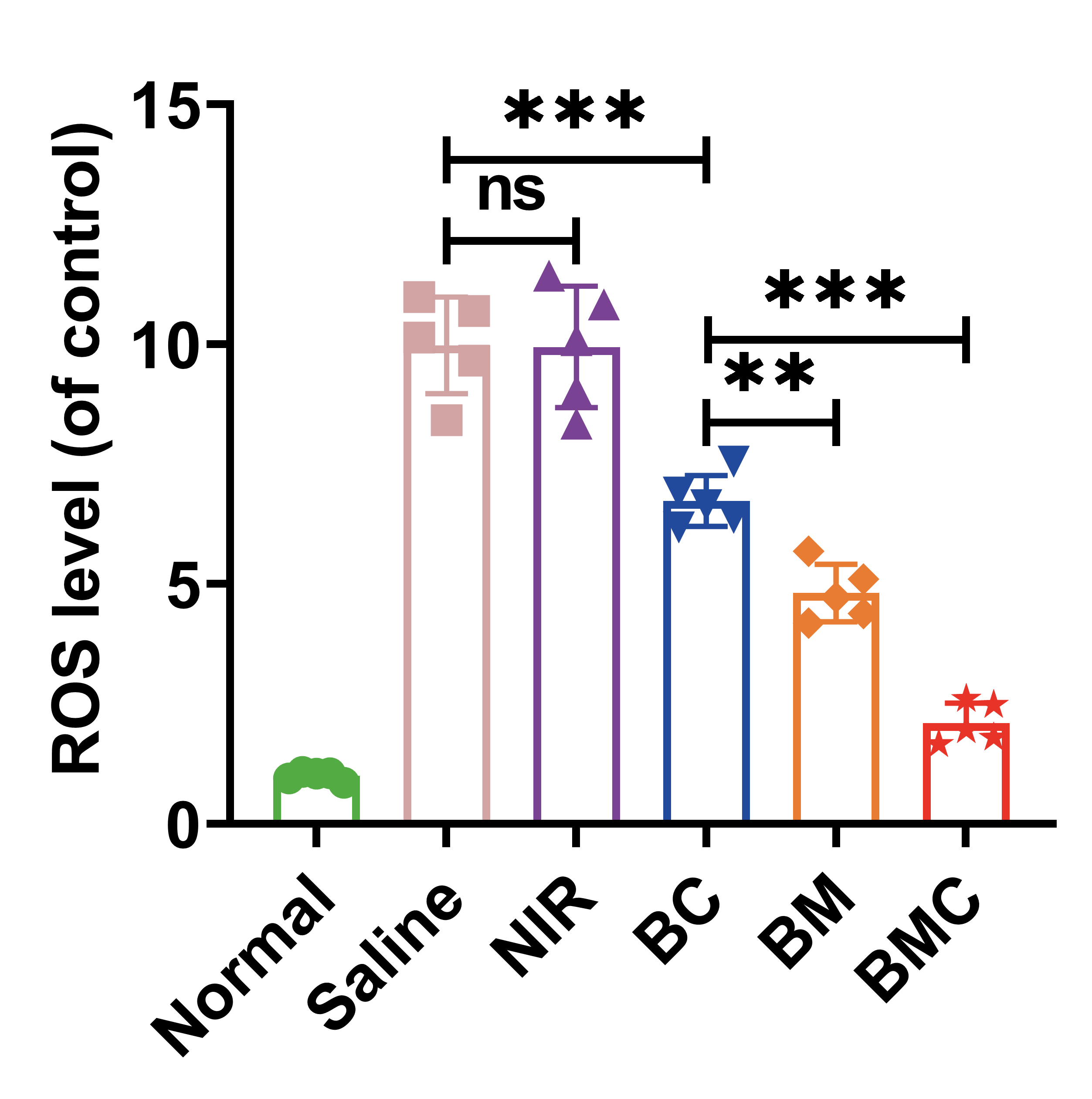


**Figure S23.** Levels of ROS in joint tissue homogenate, all data are mean ± SD, n=3, *P<0.05, **P<0.01, ***P<0.001, NS: Not Significant.


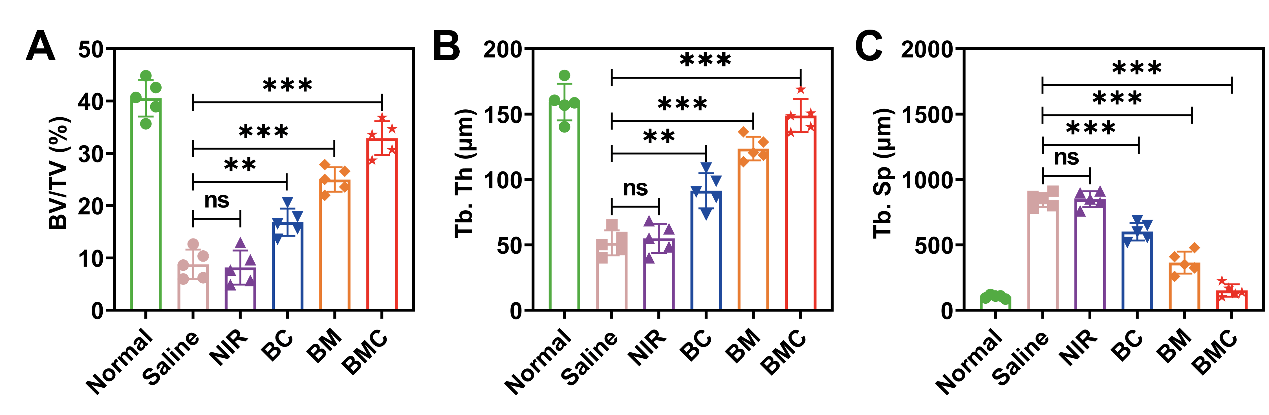


**Figure S24.** Quantitative analysis of the hind paw micro-CT data (BV, bone volume; TV, tissue volume; Tb.Th, trabecular thickness; Tb.Sp, trabecular separatio) (n=5, **P<0.01, ***P<0.001, NS: Not Significant).


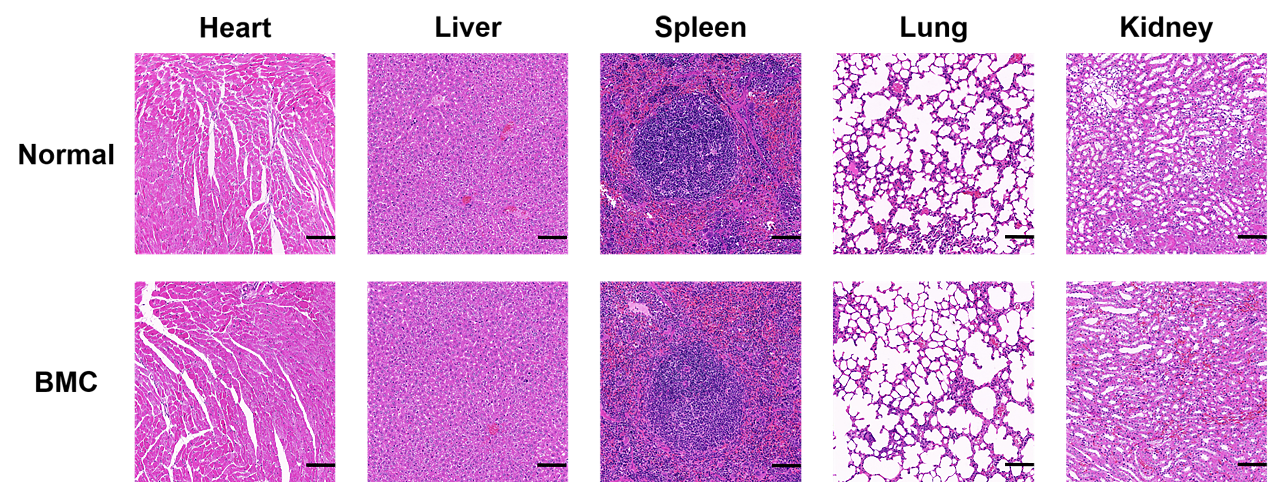


**Figure S25.** H&E staining of major organs (heart, liver, spleen, lung and kidney) of RA mice after different treatments. Scale bar is 100 μm.


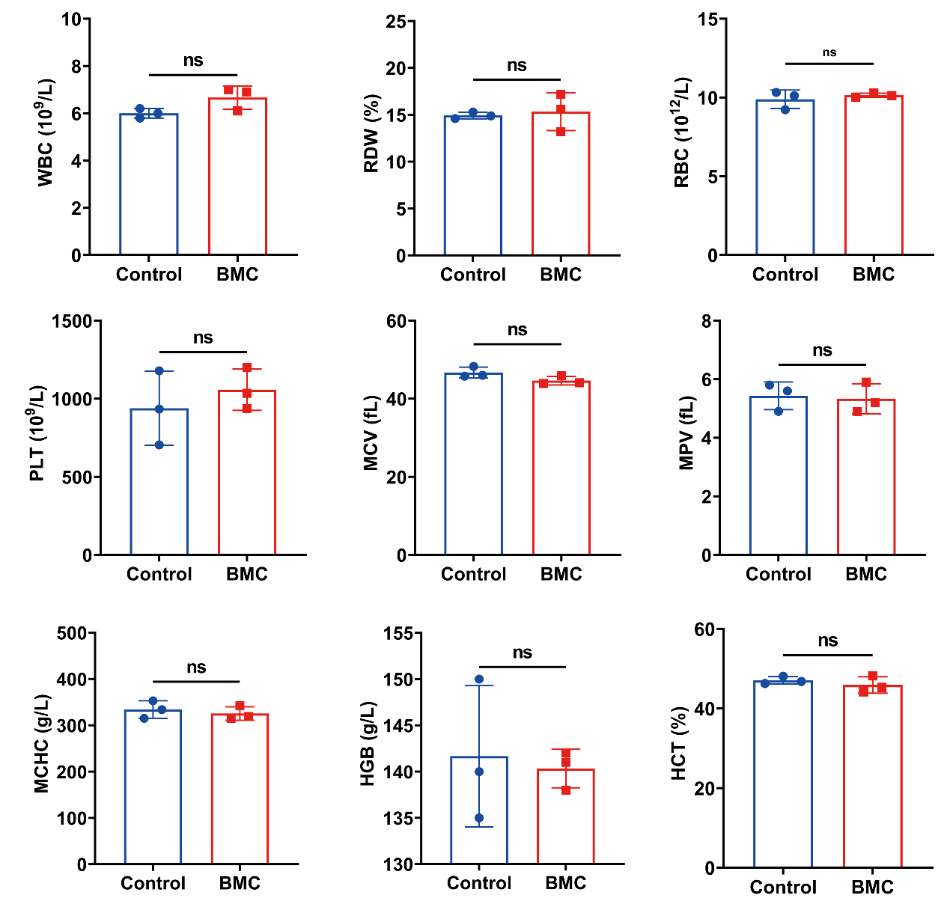


**Figure S26.** Blood routine index analysis for mice injected with saline and BMC, including MCV, MCHC, MPV, HCT, PLT and RDW (n=3, NS: Not Significant).


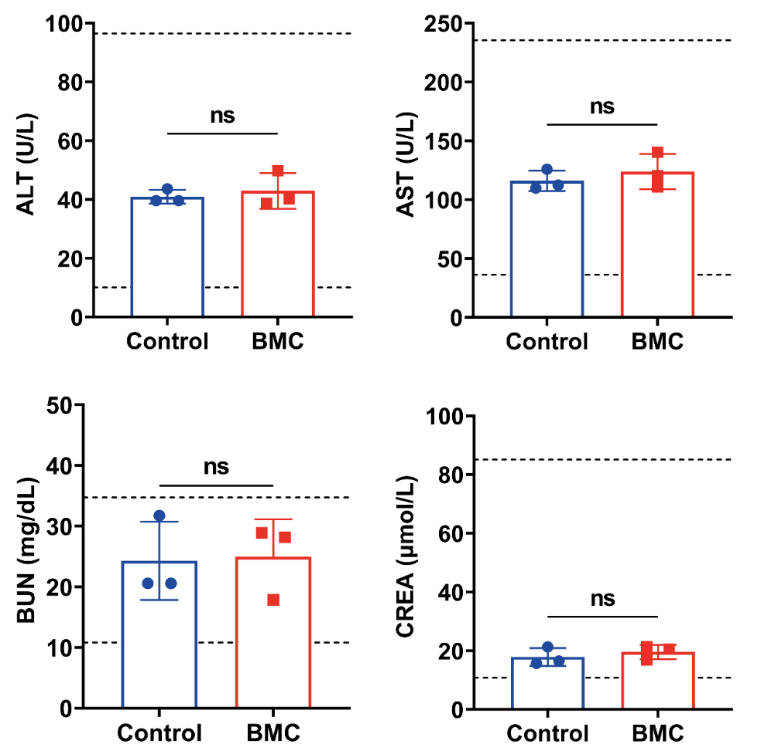


**Figure S27.** The AST, ALT, BUN, CREA level in mice after injected with BMC and saline (n=3, NS: Not Significant). The dotted lines indicate the normal range of relevant biochemical parameters in healthy mice.


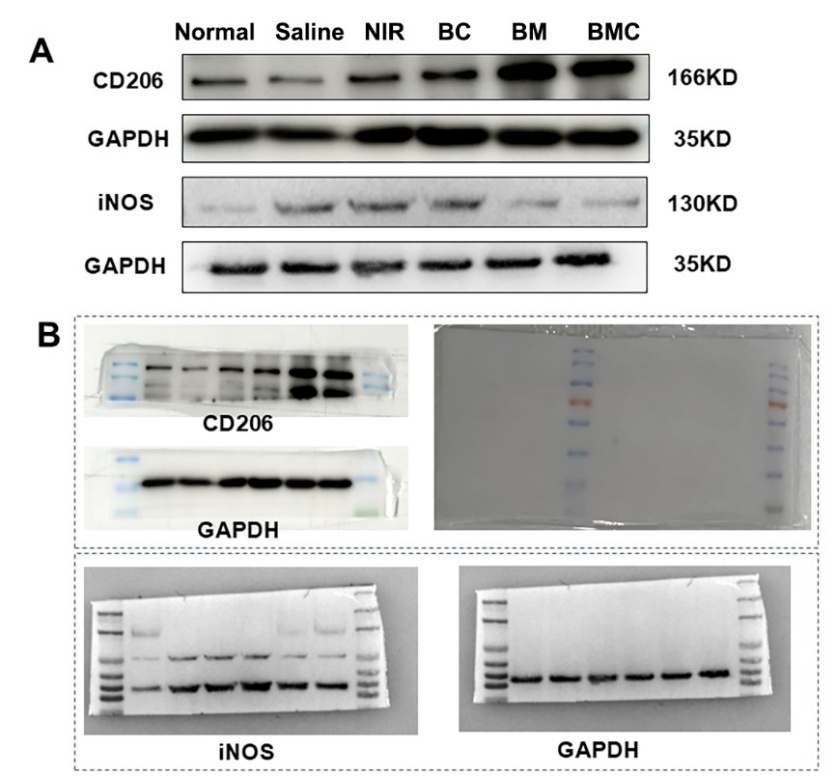


**Figure S28.** Expression of CD206 and iNOS in mice of all experimental groups investigated by western blot analysis.


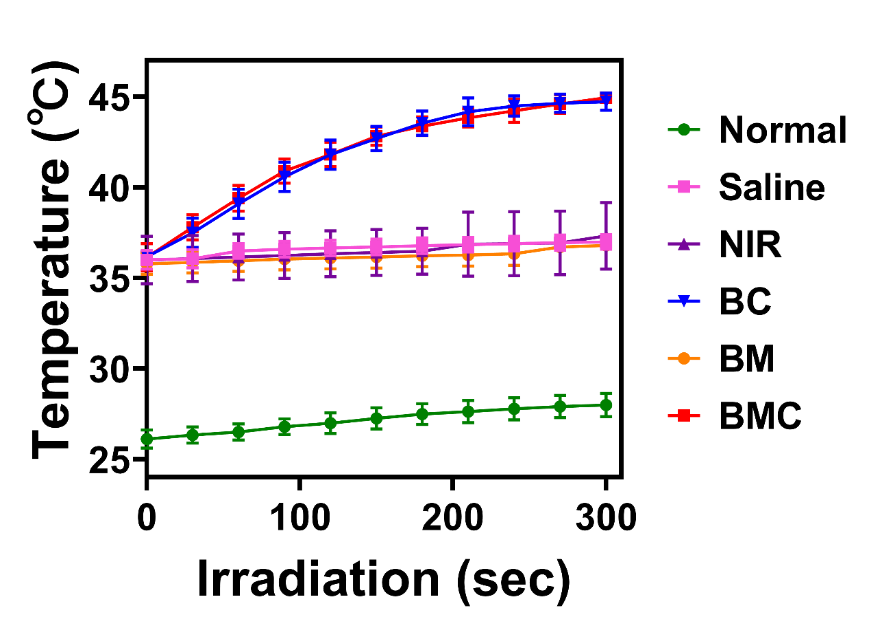


**Figure S29.** Temperature change curves of CAIA mice right paw after NIR irradiation (808 nm, 0.96 W/cm^2^) for 5 min (n=3).


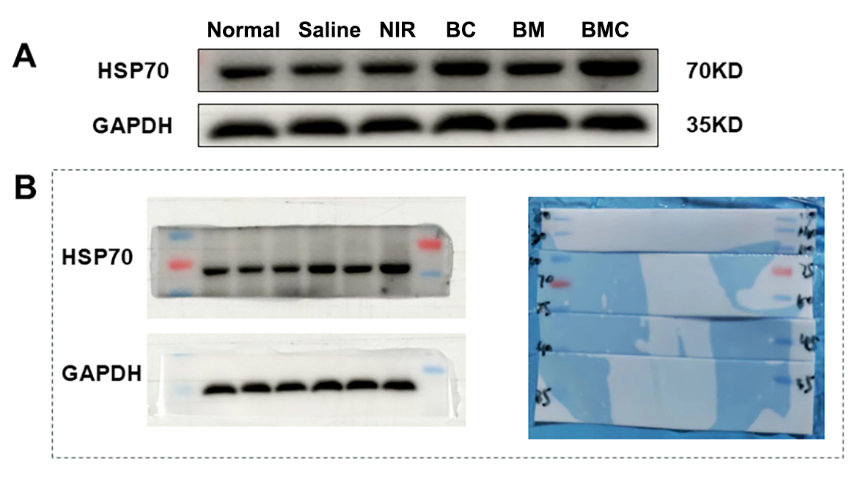


**Figure S30.** Expression of HSP70 in mice of all experimental groups investigated by western blot analysis.


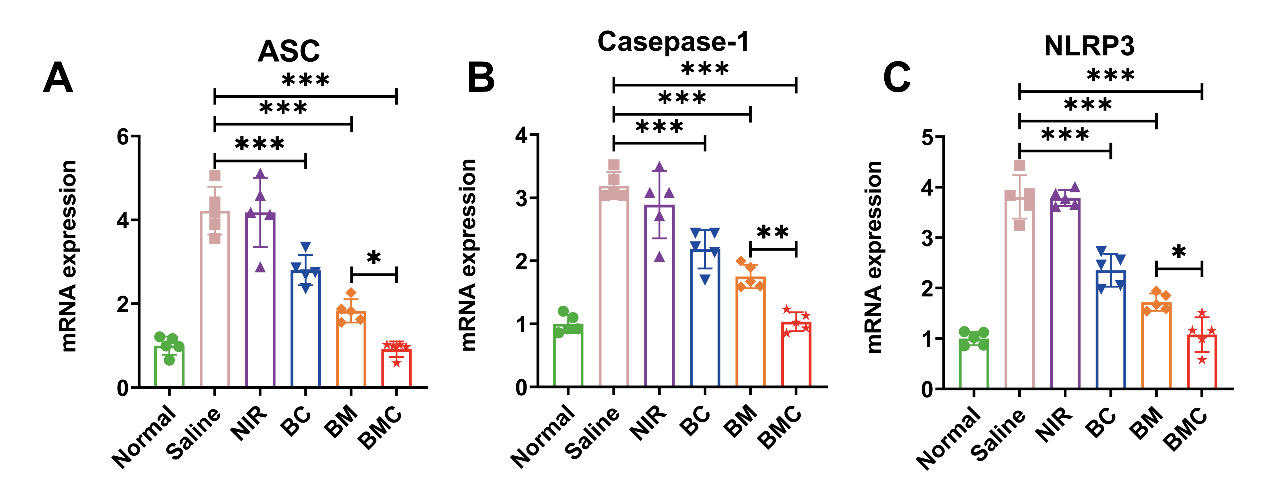


**Figure S31.** mRNA levels of NLRP 3, ASC and Caspase-1 were determined by RT-qPCR, all data are mean ± SD, n=5, *P<0.05, **P<0.01, ***P<0.001.
